# Biogenesis of the Inner Membrane Complex Is Dependent on Vesicular Transport by the Alveolate Specific GTPase Rab11B

**DOI:** 10.1371/journal.ppat.1001029

**Published:** 2010-07-29

**Authors:** Carolina Agop-Nersesian, Saskia Egarter, Gordon Langsley, Bernardo J. Foth, David J. P. Ferguson, Markus Meissner

**Affiliations:** 1 Department of Infectiology, Parasitology, University Hospital Heidelberg, Heidelberg, Germany; 2 Division of Infection & Immunity and Wellcome Centre for Parasitology, Faculty of Biomedical and Life Sciences, University of Glasgow, Glasgow, United Kingdom; 3 Laboratoire de Biologie Cellulaire Comparative des Apicomplexes, Institut Cochin, Inserm, U567, CNRS, UMR 8104, Faculté de Médecine Paris V – Hôpital Cochin, Paris, France; 4 School of Biological Sciences, Nanyang Technological University, Singapore, Republic of Singapore; 5 Nuffield Department of Clinical Laboratory Science, Oxford University, Oxford, United Kingdom; Washington University School of Medicine, United States of America

## Abstract

Apicomplexan parasites belong to a recently recognised group of protozoa referred to as Alveolata. These protists contain membranous sacs (alveoli) beneath the plasma membrane, termed the Inner Membrane Complex (IMC) in the case of Apicomplexa. During parasite replication the IMC is formed *de novo* within the mother cell in a process described as internal budding. We hypothesized that an alveolate specific factor is involved in the specific transport of vesicles from the Golgi to the IMC and identified the small GTPase Rab11B as an alveolate specific Rab-GTPase that localises to the growing end of the IMC during replication of *Toxoplasma gondii*. Conditional interference with Rab11B function leads to a profound defect in IMC biogenesis, indicating that Rab11B is required for the transport of Golgi derived vesicles to the nascent IMC of the daughter cell. Curiously, a block in IMC biogenesis did not affect formation of sub-pellicular microtubules, indicating that IMC biogenesis and formation of sub-pellicular microtubules is not mechanistically linked. We propose a model where Rab11B specifically transports vesicles derived from the Golgi to the immature IMC of the growing daughter parasites.

## Author Summary

Apicomplexan parasites belong to a group of protists known as alveolata that also includes ciliates and dinoflagellates. One of the few morphological similarities within this group is the presence of membranous sacs beneath the plasma membrane, called alveoli. In the case of apicomplexan parasites, alveoli are well developed and described as the Inner Membrane Complex (IMC) that serves as a scaffold for the machinery driving gliding motility and host cell invasion. Given the unique nature of this organelle we aimed to identify key factors that are involved in its formation. We identified a unique family of Rab11-GTPases in Alveolata. Using *Toxoplasma gondii* as a model system, we show that this small GTPase is essential for the delivery of vesicles from the Golgi to the nascent IMC of the daughter parasites. Interestingly, biogenesis of the IMC is not linked to the formation of subpellicular microtubules. We propose a model where the action of Rab11B is necessary for IMC formation.

## Introduction

The group known as alveolata unites apicomplexan parasites, dinoflagellates and ciliates to a novel infrakingdom [1]. Despite the large morphological differences between the phyla they share an endomembrane system underneath the plasma membrane comprised of membranous sacs named alveoli [2]. Apicomplexan parasites are enclosed by a pellicle comprising the plasmalemma beneath which double membraned aleveolin sacs are forming the Inner Membrane Complex (IMC). Several homologues of apicomplexan IMC-proteins, called alveolins, have been identified in other alveolates, demonstrating a common origin of the alveoli/IMC [3], [4].

The IMC plays a central role during intracellular parasite replication and as an anchor for the machinery driving gliding motility and invasion of the host cell during the extracellular state of the parasite [5], [6]. Beneath the pellicle and closely associated with it is the sub-pellicular network, which connects the pellicle to the cortical microtubules that originate from the microtubule organising centres (MTOC) at the apical tip of the parasite [7], [8]. This association results in extraordinary mechanical strength and flexibility that is further increased by the fact that the sub-pellicular microtubules are unusually stable [9].

Given the importance of the IMC as an anchor for the gliding machinery in apicomplexan parasites, research has focused on the identification and characterisation of proteins residing or anchored within the IMC (i.e. IMCs, GAPs [3], [4], [8]). However, the mechanisms involved in the biogenesis of the daughter cell IMC are unknown. Early studies have suggested that clathrin coated vesicles are generated at the single Golgi-apparatus of the parasite and subsequently delivered to the IMC [10]. However, functional loss of the Golgi-localised dynamin-related protein B (DrpB) does not result in a defect in IMC formation [11]. Duplication of the Golgi is among the earliest visible event of parasite replication and shortly precedes the onset of IMC biogenesis [12], [13]. It has been speculated that the early establishment of a polarised secretory system is a prerequisite for the formation of the daughter cell IMC [6]. As mentioned above, the IMC is in tight contact with the sub-pellicular microtubules [14] and microtubule formation is at least temporally linked to IMC biogenesis during daughter cell formation [15].

Recently it has been suggested that the actin like protein 1 (Alp1) is involved in IMC formation, since overexpression of Alp1 disrupts IMC formation in *Toxoplasma gondii* [16]. Similarly MORN1 (a protein with a membrane occupation and recognition nexus motif) has been demonstrated to localise to the basal complex and the centrocone (a unique apicomplexan structure associated with the intranuclear spindle) during daughter cell assembly [17], [18], [19]. Overexpression or deletion of MORN1 results in defects in parasite replication due to a failure in centrocone formation [17], [20].

We recently characterised the small Rab-GTPase Rab11A as an essential component of the cell division machinery. Conditional ablation of Rab11A function results in a late block in cytokinesis that also affects the late maturation of the IMC. In particular we demonstrated that late components that are anchored in the IMC, such as the glideosome, are not associated with the IMC of the daughter cells [21]. Since ablation of Rab11A function does not lead to a block in IMC biogenesis itself, other factors must exist that are required for vesicular trafficking to this unique compartment.

Since alveolins are unique to this group of organisms we speculated that a specific Rab-GTPase must be essential in this process and identified Rab11B as an alveolate specific sub-family of Rab11-GTPases. Using *T. gondii* as a model organism we found that Rab11B localises to the Golgi-apparatus in resting parasites, while in replicating parasites Rab11B accumulated at the IMC of the growing daughter parasites. Conditional ablation of Rab11B function led to a loss in IMC biogenesis, demonstrating that this GTPase is essential for the transport of vesicles to the IMC during daughter cell assembly. Nevertheless, formation of sub-pellicular microtubules still occurs in absence of Rab11B function. We present a model, summarising our current and previous findings on the molecular mechanisms involved in biogenesis and maturation of the IMC during replication of *T. gondii*.

## Results

### Rab11B a Novel Member of the Rab11 Family that is Conserved only in Alveolates

Rab-GTPases play a crucial role in the regulated delivery of vesicles from a specific donor to an acceptor compartment [22]. Given that the alveoli represent a unique organelle in the Alveolata we speculated that the presence of a unique Rab-GTPase might be the molecular basis for the evolution of this unique organelle. Therefore we searched the OrthoMCL-database (www.orthoMCL.org) for Rab-GTPases that are conserved in alveolates but are absent in other eukaryotes. The only alveolate specific Rab-GTPase identified in this search, i.e. Rab11B, belonged to orthology group OG4_21991 (Table S1). Interestingly the second Rab11 gene (Rab11A) was grouped as a regular Rab11 orthologue together with Rab11 proteins of other eukaryotes, including red algae and human. Sequence alignments suggest several unique features of Rab11B when compared to “conventional” Rab11 proteins. We identified several unique amino acid substitutions, most notably in the P-loop region that is essential for Mg^2+^ and GTP-binding and in the effector (switch) regions that contain binding motifs for Rab-associated, regulatory proteins (Figure S1) [23], [24].

**Figure 1:**
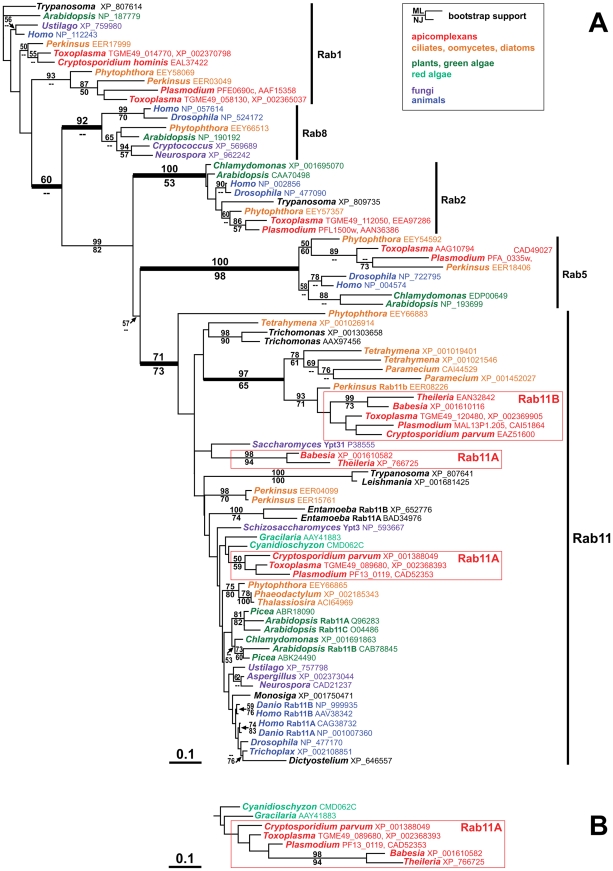
Alveolate organisms possess two distinct types of Rab11 homologues. (A) This tree, which presents the best maximum likelihood (ML) phylogeny out of 12 trees (ln likelihood = −13,028.03), clearly shows the two distinct apicomplexan Rab11 subfamilies Rab11A and Rab11B. While the Rab11B cluster includes several alveolate sequences from outside the Apicomplexa and is well supported by the bootstrap analysis, the apicomplexan Rab11A sequences are situated among the larger diversity of eukaryote Rab11 homologues, usually in close proximity to Rab11 from red algae. The apicomplexan Rab11A cluster is generally unsupported by the bootstrap analysis but does occur as a monophyletic unit in some trees: panel (B) shows the corresponding branch of another ML tree with a slightly lower likelihood (ln likelihood = −13,033.89). Bootstrap support values (100 replicates) for ML and Neighbor-Joining (NJ) analyses are indicated above and below the relevant branches, respectively, where they are greater than 50. GenBank accession numbers are indicated in the figure behind the genus names. Species names: *Arabidopsis thaliana*, *Aspergillus flavus*, *Babesia bovis*, *Chlamydomonas reinhardtii*, *Cryptococcus neoformans*, *Cryptosporidium hominis*, *Cryptosporidium parvum*, *Cyanidioschyzon merolae*, *Danio rerio*, *Dictyostelium discoideum*, *Drosophila melanogaster*, *Entamoeba histolytica*, *Gracilaria lemaneiformis*, *Homo sapiens*, *Leishmania major*, *Monosiga brevicollis*, *Neurospora crassa*, *Paramecium tetraurelia*, *Perkinsus marinus*, *Phaeodactylum tricornutum*, *Phytophthora infestans*, *Picea sitchensis*, *Plasmodium falciparum*, *Saccharomyces cerevisiae*, *Schizosaccharomyces pombe*, *Tetrahymena thermophila*, *Thalassiosira pseudonana*, *Theileria parva*, *Toxoplasma gondii*, *Trichomonas vaginalis*, *Trichoplax adhaerens*, *Trypanosoma cruzi*, *Ustilago maydis*.

We next performed a phylogenetic analysis of Rab11-GTPases that confirmed that the two Rab11-members of Alveolata differ significantly from one another (alignment can be downloaded as dataset DataS1). While Rab11A groups together with the Rab11 sequences from other eukaryotes (albeit without strong bootstrap support), Rab11B forms a strongly supported, clearly distinct clade that only contains alveolate homologues (Figure 1). Interestingly, Rab11 has undergone independent gene duplications in several taxa (i.e. plants, animals, fungi). However, the phylogenetic trees do not allow us to determine if the presence of two types of alveolate Rab11-GTPases is due to an ancestral, phylum-specific gene duplication, or if one of the two copies has been introduced via the secondary endosymbiosis of a red alga that took place during the evolution of the chromalveolate lineage [25]. Although the proximity of the red algal Rab11 sequences to alveolate Rab11A in the phylogenetic tree (Figure 1) is suggestive of the second scenario, the lack of bootstrap support for this affiliation and the absence of further evidence for a potential shared ancestry of alveolate Rab11A and red algal Rab11 leave this issue unresolved.

In summary we found that Rab11B is a unique homologue of the Rab11 family of small GTPases that can only be found in the Alveolata.

### Rab11B Cycles between the Golgi and the Inner Membrane Complex of the Daughter Parasites

**Figure 2:**
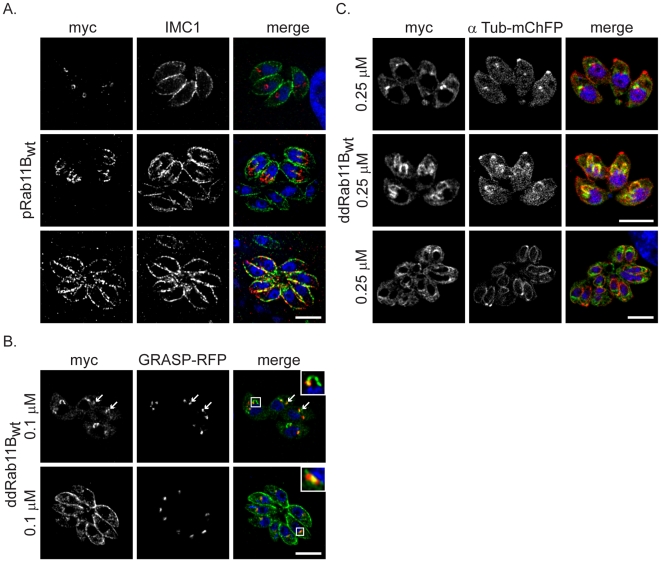
Dynamic location of Rab11B between the golgi and the nascent IMC during cell division. (A) Immunofluorescent analysis of parasites expressing the wild type Rab11B under control of the endogenous promoter (pRab11B_wt_). Parasites were double-labelled with anti-myc (red) and anti-IMC1 (green) to visualise Rab11B and IMC1. Rab11B cycles from a location close to the nucleus (interphase parasites in upper panel) to the growing IMC of the daughter parasites (dividing parasites in middle and lower panel) in a cell cycle dependent manner. (B) Immunofluorescent analysis of parasites expressing ddRab11B_wt_ and the Golgi marker (GRASP-RFP in red) in presence of 0.1 µM Shld-1 for 24 h. Parasites are labelled with anti-myc antibody (green). Rab11B localises to the Golgi at the initial phase of cell division (upper panel and inlet) and accumulates to the nascent IMC of daughter parasites during endodyogeny (see A,C middle panel). After endodyogeny is completed Rab11B again accumulates at the Golgi (lower panel and inlet). (C) Immunofluorescent analysis of parasites expressing the ddRab11B_wt_ and the α-tubulin marker (mCherry-α-Tubulin in red) in presence of 0.25 µM Shld-1 for 24 h. Parasites are labelled with anti-myc antibody (green). At the onset of endodyogeny (upper panel) Rab11B accumulates to the newly assembled conoid of the daughter cells. Later becomes concentrated along the daughter scaffold throughout endodyogeny (middle and lower panel). Scale bars represent 5 µm.

**Figure 3:**
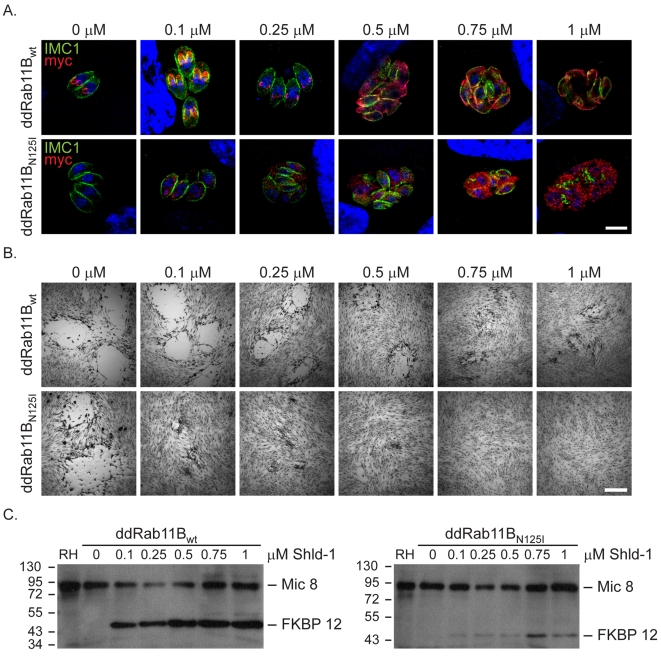
Functional loss of Rab11B results in the disruption of the daughter cell IMCs. Analysis of parasites expressing an ectopic copy of ddRab11B_wt_ or ddRab11B_N126I_. (A) A Shld-1 concentration gradient with the clonal ddRab11B_wt_ (upper panel) and ddRab11B_N125I_ (lower panel) using immunofluorescent analysis. Parasites were double-labelled with anti-myc (red) to visualise Rab11B and anti-IMC1 (green). Parasites expressing ddRab11B_N125I_ show a severe defect on the IMC formation at low Shld-1 concentration. In contrast, ddRab11B_wt_ expressing parasites demonstrate a relative weak phenotypic deformation of the IMC at high Shld-1 concentrations. Scale bar represents 5 µm. (B) Growth analysis of the indicated parasites grown on HFF cells for 7 days. In the ddRab11B_wt_ expressing parasites plaque size is slowly reduced with increasing Shld-1 concentrations (upper panel). In the dd-Rab11B_N125I_ parasites growth is strongly inhibited at low concentrations of Shld-1 and completely absent at concentrations <0.5 µM Shld-1 (lower panel). Scale bar represents 5 mm. (C) Immunoblot analysis of clonal ddRab11B_wt_ (left) and ddRab11B_N125I_ (right) transfectans. Rising levels of the fusion proteins (40 kDa) can be detected in presence of increasing Shld-1 concentrations. For detection of Rab11B the blot was probed with anti-FKBP12 (Affinity BioReagents). MIC8 served as internal loading control. Note that in presence of 0.1 µM Shld-1 ddRab11B_wt_ is almost fully stabilised, whereas ddRab11B_N125I_ is barely detectable.

To analyse the role of Rab11B, we used *Toxoplasma gondii* as a model system. Since the location of most Rab-GTPases is determined by geranylation of C-terminal cysteines, we decided to express N-terminally tagged versions of Rab11B. Therefore we generated two constructs for the localisation study and generated stable transfected parasites. The first construct allowed expression of a myc-tagged version of Rab11B under control of its own promoter region (pRab11B) to ensure correct timing of expression. The second construct allowed expression of a ddFKBP-tagged copy of Rab11B and therefore tuneable control of the protein level [26], which minimises the risk of overexpression phenotypes at low inducer concentrations. We found identical location and behaviour of the transgenic Rab11B-versions (Figure 2 and Figure 3A).

The location of Rab11B was highly dynamic and strongly dependent on the cell cycle of the parasite. In resting parasites (prior to the occurrence of the daughter cell IMCs) Rab11B was concentrated at the apical side of the nucleus (Figure 2A,B,C upper panels). During cell division Rab11B accumulated at the growing IMC of the developing daughter cells (Figure 2A,C middle panels). This accumulation was observed until endodyogeny of the parasites has been completed and daughter parasites bud out from the mother cell (Figure 2A,B lower panels).

We confirmed that in resting parasites the observed accumulation of Rab11B close to the nucleus corresponds to the single Golgi-stack [13], [12] as demonstrated by co-localisation with the Golgi-marker GRASP-RFP [27], while in dividing parasites Rab11B accumulated with the IMC of the developing daughter parasites (Figure 2B,C).

Likewise, in parasites co-expressing ddRab11B_wt_ and mCherry-tagged alpha-Tubulin we found that Rab11B co-localised with the nascent IMC of the daughter cells at the onset of endodyogeny (Figure 2C).

In conclusion our localisation study indicated that Rab11B is cycling between the Golgi and the IMC of the forming daughter cells and strongly suggested a role of Rab11B in the vesicular transport of vesicles from the Golgi to the IMC.

### Expression of a Dominant-Negative Rab11B

During the delivery of vesicular material from a donor- to an acceptor-membrane Rab-GTPases cycle between the different compartment [28]. In order to characterise the function of Rab proteins different strategies, including overexpression and expression of dominant negative versions of the respective Rab-protein have been employed (see for example [29], [30]).

To functionally analyse the role of Rab11B we generated a construct for the expression of a dominant negative version of Rab11B. Therefore a point mutation was introduced in the GTPase domain at position 125 exchanging an asparagine to isoleucine that locks the protein in the GDP bound form [21] (supplementary Figure S1). We generated parasites stably expressing ddRab11B_N125I_ and confirmed that the protein can be efficiently regulated in dependence of Shld-1 as evident on immunoblot and immunofluorsecence microcopy (Figure 3A,C). As expected the GDP-locked Rab-protein featured a rather vesicular pattern in the cytosol of the parasite (Figure 3A, 4A). The ddFKBP-system allows a rapid stabilisation of protein levels and we previously demonstrated that upregulation of dominant negative Rab11A in extracellular parasites results in a significant decrease in host cell invasion [21], [26]. In contrast, upregulation of dominant negative Rab11B did not have any effect on the ability of the parasites to invade the host cell (data not shown). Instead we found that stabilisation of ddRab11B_N125I_ results in a block in intracellular growth of the parasite with a strong phenotypic defect on the formation of the IMC (Figure 3A and Figure S2). The defect is consistent with almost complete growth inhibition with the absence of visible plaques in the growth assay (Figure 3B lower panel). Interestingly, nuclear division took place in parasites expressing the dominant negative Rab11B, while IMC formation is completely disrupted ultimately leading to the formation of multinucleated cells (supplementary Figure S2, Figure 3A). Prolonged growth in the presence of the ligand subsequently leads to a collapse of the mother cell. In contrast parasites expressing wild type Rab11B showed a relatively mild effect on IMC biogenesis that can only be detected at a high ligand concentration (>0.5 µM, Figure 3A,B). The results are consistent with the determined protein level from whole – cell – extract on western blot, which indicated that the wild-type protein can be expressed at much higher concentrations compared to the dominant – negative protein. In case of the wild type protein maximal stabilisation can be reached already at low inducer concentration (∼0.1 µM Shld-1), when no phenotype can be observed. In comparison the dominant negative version of Rab11B is barely detectable at inducer concentrations that lead to a complete growth inhibition (∼0.1 µM Shld-1; Figure 3C).

**Figure 4:**
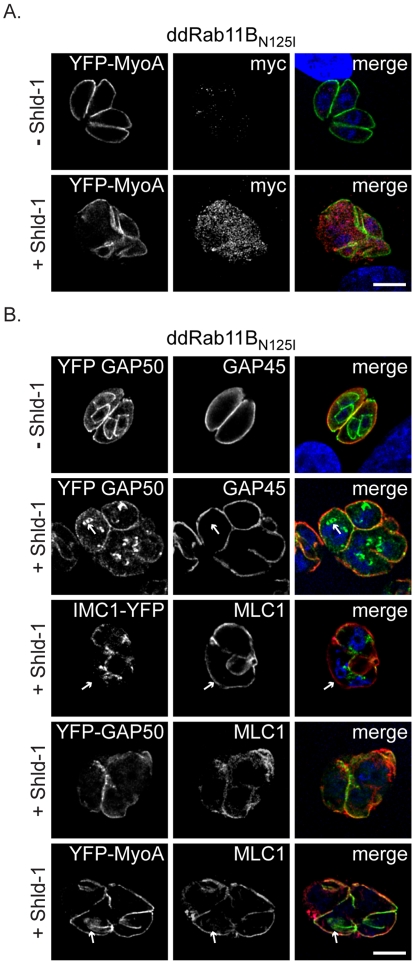
Phenotypic analysis of the IMC formation in dd-Rab11B_N125I_ expressing parasites. (A) ddRab11B_N125I_ parasites co-expressing YFP-MyoA (green) were treated without and with 1 µM Shld-1 for 24 h. For detection of the dominant negative Rab11B, parasites were labelled with anti-myc (red). (B) ddRab11B_N125I_ parasites co-expressing different YFP-tagged components of the glideosome/IMC (green) were treated without and with Shld-1 for 24 h. In absence of Shld-1 Gap50 can be identified in the IMC of first- and premature second-generation daughter parasites, while Gap45 can only be detected in the mature IMC (upper panel). In presence of Shld-1, early and late components appear comparably affected during IMC assembly. Initial nucleation of the daughter IMC can still take place (white arrow, second panel). MLC1 remains restricted to the pellicle of the first generation mother cell, while only a faint staining of IMC 1 can be detected (white arrow, middle panel). MLC 1 and MyoA only co-localise at the IMC of the mother, while MLC1 also accumulates close to the nucleus of forming daughter cells (white arrow, last panel). Scale bars represent 5 µm.

### Rab11B is Essential for Early Steps in the Biogenesis of the Daughter IMC

In the developing daughter cells the biogenesis of the IMC proceeds in a stepwise process associated with a lateral growth and a subsequent maturation comprising the final assembly of the gliding machinery [21], [31]. To define the step of IMC biogenesis that requires Rab11B, we investigated the fate of different components of the IMC, such as the early components IMC1 (a structural element of the sub-pellicular network [8]) and the gliding associated protein 50 (GAP50) (an anchor protein for the pre-assembled glideosome complex, [31]) and late components such as the proto-glideosome associated proteins (GAP45, MyoA and MLC1; [31]). We found that stabilisation of ddRab11B_N125I_ results in mislocalisation of early components during IMC assembly. This apparent lack of IMC biogenesis results in multinucleated parasites that maintained the IMC of the original mother cell (Figure 4B, see signal for IMC1 and GAP50). Similarly, late components of the IMC were only identified at the IMC of the mother cell (Figure 4A,B). Moreover, in case of the motor protein MyoA and its light chain MLC1, we observed several cases, where MLC1 is associated with filament like structures that lack a signal for MyoA (Figure 4A, lower panel).

**Figure 5:**
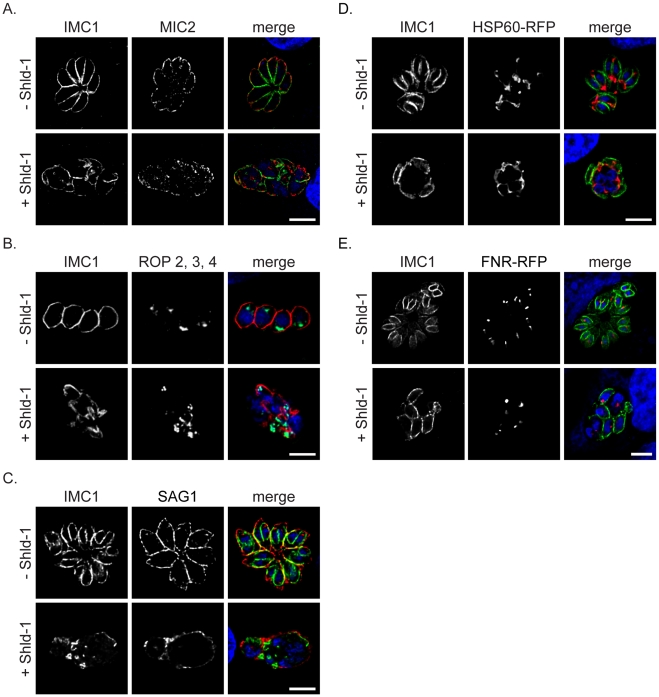
Fate of the secretory organelles and mitochondrial division in dd-Rab11B_N125I_ expressing parasites. (A,B and C) Immunofluorescent analysis of ddRab11B_N125I_ expressing parasites incubated in absence and presence of Shld-1 for 24 h and stained with the indicated antibodies. In presence of Shld-1 the organelles are still formed, although the organelles lose their apical localisation (see also Figure 7, 8). SAG1 appears to be correctly localised to the plasma membrane of the mother parasite indicating functional transport of GPI-anchored proteins to the surface.(D and E) ddRab11B_N125I_ parasites co-expressing the mitochondrial marker HSP60-RFP (red) or the apicoplast marker FNR-RFP (red) were treated without and with Shld-1 for 24 h and subsequently labelled with anti-IMC1 (green). In presence of Shld-1 mitochondrial division and apicoplast segregation appeared normal. Scale bars represent 5 µm.

We were also interested if other steps are affected during the intracellular development of the parasite, such as biogenesis of the apically localised secretory organelles (micronemes and rhoptries). Although biogenesis of these organelles appeared to take place in absence of IMC formation, we observed several cases where the organelles were more randomly distributed within the parasites, indicating a loss of cell polarity due to the apparent lack of IMC biogenesis (Figure 5A, B). We also confirmed that SAG1 (surface antigen 1) is normally localised in the plasmamembrane of the mother cell (Figure 5C).

In addition, we did not observe significant effects on the duplication and segregation of mitochondria or apicoplast (Figure 5D,E). Together these data demonstrate that Rab11B is exclusively required for the formation of the IMC.

### Expression of Rab11B_N125I_ does not Affect Segregation of the Golgi and Formation of the Sub-pellicular Microtubules

**Figure 6:**
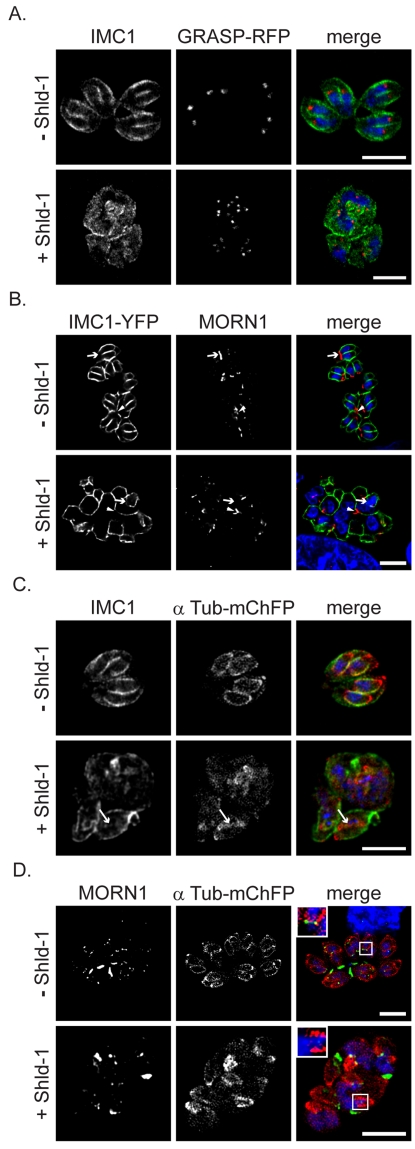
Absence of IMC biogenesis does not interfere with formation of sub-pellicular microtubules. (A) ddRab11B_N125I_ parasites co-expressing the Golgi marker GRASP-RFP (red) were treated without and with Shld-1 for 24 h and labelled with the indicated antibodies (green). In presence of Shld-1 no IMC is formed, but nuclear and Golgi division appears normal (B) ddRab11B_N125I_ parasites co-expressing IMC1-YFP (green) were grown in absence and presence of Shld-1 for 24 h and subsequently labelled with anti-MORN1 (red). In presence of inducer the centrocone close to the nucleus appears normal (arrow head). However, disruption of IMC formation inhibits formation and relocalisation of the posterior polar ring of daughter cells (white arrow). (C and D) ddRab11B_N125I_ parasites co-expressing mCherry-α-Tubulin (red) were treated without and with inducer for 24 h and labelled with the anti-IMC1 antibody (green) or with anti-MORN1 antibody (green), respectively. Abrogation of IMC formation does not inhibit assembly and growth of the sub-pellicular microtubules (white arrow). Nevertheless, no formation of the basal complex can be observed despite presence of initial daughter formation (see inlet). Scale bar represents 5 µm.

So far our analysis indicated that Rab11B plays a role in the transport of vesicles from the Golgi to the nascent IMC of the daughter cells. Since biogenesis of secretory organelles appeared to be unaffected (Figure 5A,B), we predicted that expression of Rab11B_N125I_ has no direct effect on the Golgi apparatus. In fact, segregation of the Golgi appeared normal, since each nucleus was associated with a Golgi apparatus (Figure 6A). To examine whither steps upstream of IMC formation might be affected, we analysed the location of MORN1, a marker of the centrocone/nuclear pole and the posterior polar ring of the pellicle of the mother cell [17]. The typical location of MORN1 at the centrocone and posterior pore of the mother cell appeared relatively normal. However, the abrogation of IMC biogenesis appeared to block the development and relocation of the posterior polar rings normally associated with daughter cell formation, resulting in multinucleated parasites with MORN1 positive centrocones and absence of formation of posterior polar rings in the cytoplasm (Figure 6B,D). Interestingly, we found that abrogation of IMC biogenesis did not lead to a block in formation of the sub-pellicular microtubules. Thus, elongation of the sub-pellicular microtubules appears independent from the formation of the daughter IMC (Figure 6C,D and see below). We verified that in absence of IMC biogenesis nascent sub-pellicular microtubules were not associated with a MORN1 ring (Figure 6B,D).

Together these results demonstrate that the maturation and formation of the posterior polar ring is dependent on IMC biogenesis, while segregation of the centrocone and Golgi and polymerisation of sub-pellicular microtubules is independent of vesicular transport from the Golgi to the IMC of the forming daughter cells.

### Ultrastructural Analysis of Dominant-Negative Expressing Rab11B

**Figure 7:**
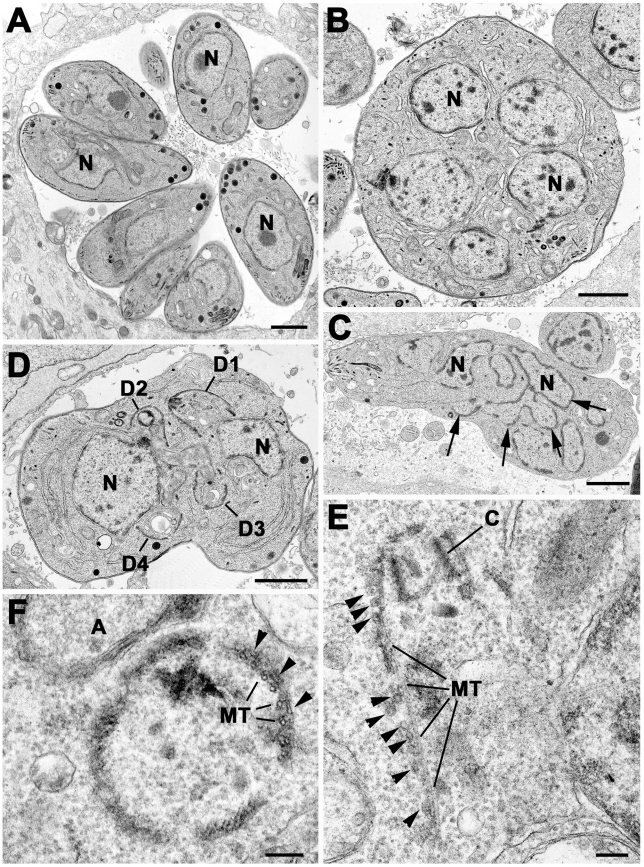
Electron micrographs of sections of *T. gondii* in fibroblast. Parasites were grown without (A) or with Shld-1 (B–E) for 36 h. (A) Low power through a parasitophorous vacuole showing a rosette of eight parasites having undergone three cycles of repeated endodyogeny. N – nucleus. (B) Low power of a large spherical parasite that appears to have a number of nuclei (N) but no evidence of daughter formation. (C) Section through a slightly elongated parasite that appears to have a complex nuclear structure (N) with a number of lobes (arrows). (D) Section through a parasite with two nuclei (N) in which four conical structures representing the initiation of daughter formation (D1–4) can be identified. In A–D scale bar represents 1 µm. (E) Detail showing an early daughter from a stage similar to that in D. The apical conoid (C) and longitudinally running sub-pellicular microtubule (MT) can be identified. Note the absence of IMC on the outside of the microtubule although a number of vesicles (arrows) could be identified. (F) Detail of a cross section through an early daughter showing the sub-pellicular microtubules (MT) with overlying electron dense material (arrows) and an absence of the IMC. A – apicoplast. In E and F scale bar represents 100 nm.

**Figure 8:**
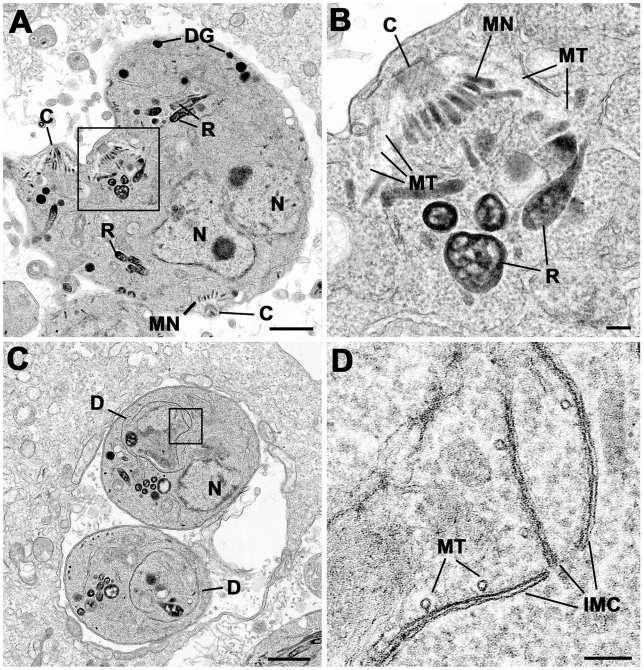
Electron micrographs of parasites cultured with Shld-1. Parasites were cultured for 24 h (C, D) and 36 h (A, B) Prior to Fixation. (A) Low power of sub-spherical parasite showing multiple nuclei (N) and abnormally located conoids (C) and groups of rhoptries (R) and micronemes (MN) within the mother cell cytoplasm. Scale bar represents 1 µm. (B) Detail of the enclosed area in (A) showing the conoid and sub-pellicular microtubules (MT) representing the anterior of a daughter. There are normal appearing rhoptries (R) and micronemes (MN) associate with the apex of the daughter but note the absence of an overlying IMC. Scale bar represents 100 nm. (C) Lower showing two parasites that appear to be undergoing endodyogeny in which the developing daughters (D) can be identified. N – nucleus. Scale bar represents 1 µm. (D) Detail of the enclosed area in C showing the presence of the IMC and underlying microtubules (MT) but note the abnormal gaps and overlapping between the plates of the IMC. Scale bar represents 100 nm.

To verify the results obtained from light microscopy we performed ultrastructural analysis of ddRab11B_N125I_ expressing parasites. Parasites were grown in presence or absence of Shld-1 and analysed after 24 and 36 hours, as indicated (Figure 7 and 8).

At both time points, the samples not treated with Shld-1 possessed large parasitophorous vacuoles (PV) with multiple daughters (2–16) showing evidence of repeated endodyogeny (Figure 7A). In contrast, the parasites in the Shld-1 treated samples display a range of features. Most PVs contain few parasites (2–4) and a high proportion of these displayed abnormal features. Many of the parasites were enlarged and more spherical in appearance (lost their polarised shape, Figure 7B,C,D). In a number of parasites there appeared to have been nuclear division with multiple nuclei distributed through the cytoplasm with no evidence of the initiation of daughter formation (Figure 7B). However, in others there was a large nucleus with an elongated and lobated appearance (Figure 7C). In addition, in many parasites there was evidence of the early stages of daughter formation (Figure 7D). Unlike normal endodyogeny with the initiation of two daughters, there was evidence for the simultaneous initiation of up to eight daughters associated with the multiple nuclei (Figure 7D). These were identified by the formation of conical structures with a central conoid (Figure 7D,E). However, on closer examination, these daughter anlagen were abnormal in lacking the double unit membranes (Figure 7E,F) of the IMC but in certain cases it was possible to identify what appear to be unfused vesicles located along the anlagen (Figure 7E). This was slightly variable and certain parasites did have some remnants of the IMC (Figure 8C,D). In all cases it was possible to identify the sub-pellicular microtubules running posteriorly from the conoid forming the typical conical shape associated with early daughter formation (Figure 7E,F,8A,B). There was also evidence of some electron dense material which may represent the sub-IMC cortical material (Figure 7F). The sub-pellicular microtubules of these atypical anlagen stretched for a short distance and could be seen partial enclose the apicoplast but not the nucleus. In certain parasites, it was noted that typical bulbous rhoptries and micronemes, not normally present in early daughters, could be associated with these structures (Figure 8A,B). However, due to the limited nature of daughter formation, these multiple apical complexes were randomly distributed throughout the cytoplasm, which would explain the immuno-fluorescent images (Figure 5B and 8A). The apicoplast appeared normal and had undergone division. In fact the division of the nucleus and the simultaneous formation of multiple daughters and their location are similar to that undergone during endopolygeny -prior to IMC biogenesis- in the coccidian stages in the cat intestine (compare Figure 7B and D with 5C in [48] and Figure 2A in [19] respectively).

## Discussion

The three layered pellicle consisting of the plasma membrane and the alveolar sacs is a unique structure in Alveolata [2]. This system plays a crucial cytoskeletal role in apicomplexan parasites, where the so called IMC has not only a structural function but is also crucial for the special mode of locomotion, called gliding motility [5] and the specialised form of intracellular cell division. There are three diverse mechanisms of cell division in Apicomplexa termed schizogony, endopoly- or endodyogeny in respect to time point of karyokinesis and cytokinesis [6], [19]. In all cases a major process during cell division involves the biogenesis of the scaffolding IMC/cytoskeleton complex. Once the replication of the daughter parasites is initiated the *de novo* synthesis of the apical organelles and the fission of the endosymbiotic organelles take place [12], [18].

During all these steps the parasite has to efficiently coordinate and control its vesicular traffic to ensure proper timing of organelle biogenesis and proper transport of the respective cargo to the nascent organelles [32]. The importance of vesicle trafficking for the formation and maturation of the IMC has been previously suggested based on ultrastructural studies that implicated Golgi-derived, clathrin coated vesicles in IMC biogenesis [10]. In fact our own data support this observation, since a mutant parasite for the clathrin heavy chain (CHC1) is not capable of forming an IMC or secretory organelles (Breinich et al., in preparation).

Given the fact that the Alveolata evolved unique organelles linked to the secretory traffic, we speculated that an alveolate specific trafficking factor must be responsible for the vesicular transport to these organelles. We recently demonstrated that an alveolate specific mechanoenzyme, the dynamin-related-protein B (DrpB), is essential for the biogenesis of the specialised secretory organelles (micronemes, rhoptries and dense granules) [11].

Here we identified the alveolate specific Rab-GTPase, Rab11B as essential for IMC formation during endodyogeny in *T. gondii*. The genomes of these organisms contain two types of Rab11 homologues, with the Rab11B gene apparently being present in multiple almost identical copies in some taxa like for example the ciliate *Paramecium* (e.g. accession numbers CAI44564 and CAI44529) and the oyster pathogen *Perkinsus* (e.g. EER08226 and EER20492). Interestingly, Rab11 genes have been duplicated independently also in several other phyla with multiple copies being found for example in plants, vertebrates and the protozoans *Trichomonas* and *Entamoeba* (Figure 1; see also e.g. [33], [34]), which is similar to the situation found for other trafficking factors, such as Rab5 [35], [36]. However, in the case of alveolate Rab11 we found that the sequences comprising the two groups Rab11A and Rab11B are highly distinct and have been derived either by a gene duplication event in an early ancestor of the alveolates, or, as one may argue, by the secondary endosymbiotic uptake of a red alga, in agreement with the chromalveolate hypothesis [25]. However, in the absence of further corroborating evidence our analyses cannot definitely determine the origin of the alveolate Rab11 homologues. *T. gondii* Rab11B is specifically required for the biogenesis of the IMC during replication of the parasite. We demonstrated that Rab11B shows a highly dynamic, cell cycle dependent location between the Golgi and the IMC of forming daughter parasites.

Functional loss of Rab11B resulted in a specific defect in the morphology and the assembly of the IMC. Surprisingly, we found that a block of IMC membrane biogenesis has no direct effect on the formation of conoid or sub-pellicular microtubules of the daughter parasites. In contrast, treatment of intracellular parasites with the plant herbicide oryzalin causes a block in polymerisation of sub-pellicular microtubules and subsequently blocks IMC formation [37]. Therefore it appears that IMC formation depends on the formation of sub-pellicular microtubules as a scaffold but not vice versa. Indeed we observed several vesicles accumulated at the sub-pellicular microtubules in the Rab11B mutant, indicating that vesicles are still transported to their destination, but are unable to fuse in order to form the nascent IMC membrane of the daughter cells (see Figure 7E).

**Figure 9:**
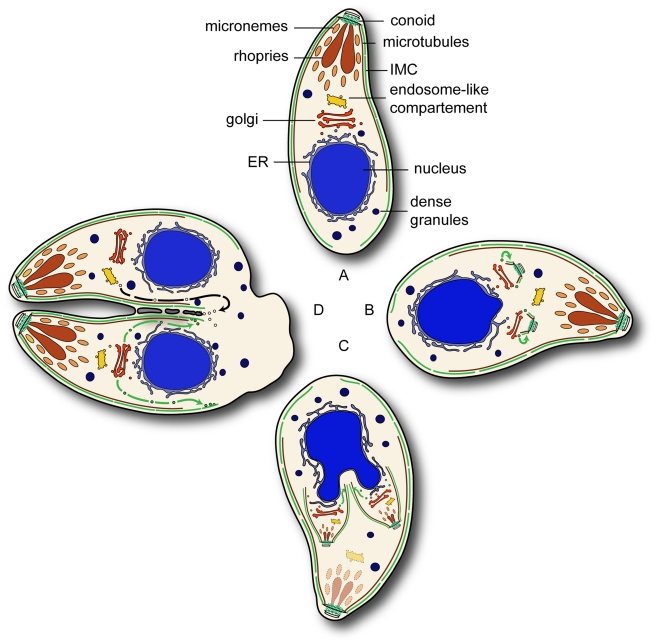
Overview of the Rab11 functions during endodyogeny. (A) In interphase parasites Rab11B resides at the Golgi. (B) During the initial phase of endodyogeny duplication of the Golgi and the formation the apical complex takes place and subsequently the nucleation of the IMC. (C) In developing daughter cell Rab11B cycles between Golgi and the nascent IMC of the daughter. (D) In the final step of cytokinesis separation of the two daughter cells requires the formation of novel plasma membrane by Rab11A dependent vesicular transport. The green and black arrows indicate Rab11B and Rab11A mediated transport respectively.

In summary we propose that an alveolate specific Rab GTPase Rab11B is required for the biogenesis of the IMC. In the interphase parasites Rab11B resides at the Golgi (Figure 9A). Contemporaneously to the segregation of the Golgi the polar ring becomes tethered with the Golgi which provides the membrane source for the nascent IMC. Additionally, the polar ring serves as MTOC responsible for assembly of the conoid and template for the polymerisation of the cortical microtubules. At the same time relocalisation of Rab11B is initiated to transport the Golgi-derived vesicles to induce nucleation of the daughter IMC (Figure 9B). Furthermore, Rab11B appears responsible for the delivery of the alveolar vesicle to provide sufficient membrane material for the on-growing IMC scaffold (Figure 9C). After completion of IMC formation, Rab11A-mediated delivery of vesicular cargo to the plasma membrane occurs which is important for completion of cytokinesis, similar to its role in other eukaryotes (Figure 9D and [21], [38]). During this process late components of the glideosome are delivered to the IMC [21].

## Materials and Methods

### Generation of Constructs

For the pRab11BmycRab11B-HXGPRT construct the 5′ UTR of Rab11B was amplified from genomic DNA RH *Δhxgprt* parasites using the oligo set pRab11B-sense (5′ CGGGGTACCAGTCCATCCCGTCTTTTGTGCATC) and pRab11B-antisense (5′ CCGGAATTCGAAAAACGACTTTTTCCGCTTTACAAGAGAGC). The fragment was introduced into the pRab11AmycRab11A-HXGPRT plasmid [21] by replacing the Rab11A promoter through the KpnI and EcoRI restriction sites. In a second step the full length cDNA of Rab11B was amplified using the oligo set Rab11B-sense (5′ GCGATGCATGGGGGTTCTGAAGACTACG) and Rab11B antisense (5′ GCGTTAATTAACCACAGCAGGACAGATTCTGAGGG) and placed downstream of the myc-tag using the NsiI and PacI restriction sites.

To place Rab11B under the control of the ddFKBP – system, the full length cDNA was introduced into the p5RT70DDmycGFP-HXGPRT plasmid [26] by replacing the GFP fragment through the NsiI and PacI restriction sites. To generate the dominant negative Rab11B construct the point mutation N125I was introduced into the wild type Rab11B cDNA using a mutated oligo Rab11B-N125I (5′ GGTTGGGATCAAACTCG) and the megaprimer method described previously [39]. The mutated cDNA fragment was subsequently introduced into the p5RT70DDmycGFP-HXGPRT plasmid [26] by replacing GFP fragment through the NsiI and PacI restriction sites.

For the co localisation studies the following fusion proteins were transfected YFP-MyoA [40], YFP-GAP50 [31], IMC1-YFP [15], FNR-RFP ([27], GRASP-RFP [27], HSP60-RFP [41] and mCherry - α Tubulin (kind gift from Borris Striepen).

### *T. gondii* Cultivation and Transfection

*T. gondii* parasites (RH *Δhxgprt*) were grown in human foreskin fibroblast (HFF) and maintained in Dulbecco's modified Eagle's medium (DMEM) supplemented with 10% fetal calf serum, 2 mM glutamine and 25 µg/ml gentamycine. For the generation of stable transformants, 5×10^7^ freshly released RH *Δhxgprt* parasites were transfected by electroporation with 30 µg of linearized plasmid DNA [42] and selected in presence of 25 µg/ml mycophenolic acid and 40 µg/ml xanthine as previously described [43]. The selection of the fusion proteins was based on 1 µM pyrimethamine or 20 µM chloramphenicol acetyltransferase (CAT) described previously [44];[45].

### Immunofluorescence Analysis

For immunofluorescence analysis, HFF cells grown on coverslips were inoculated with *T. gondii* parasites in absences or presence of 0.1–1 µM Shld-1 for 24 h. Cells were fixed either with 4% w/v paraformaldehyde in phosphate buffered saline (PBS) for 20 min at room temperature or −20°C cold methanol for 5 min, respectively. Fixed cells were permeabilized with 0.2% Triton X-100 in PBS for 20 min followed by blocking in 2% w/v bovine serum albumin (BSA) in PBS for 20 min.

The staining was performed using different combinations of primary antibodies for 60 min and followed by secondary Alexa Fluor 488 or Alexa Fluor 594 conjugated antibodies for another 60 min, respectively (1∶3000, Invitrogen-Molecular Probes).

For image acquisition z-stack of 0.15 µm increments were collected on a PerkinElmer Ultra – View spinning disc confocal Nikon Ti inverted microscope equipped with a Hamamatsu EM – charged coupled device (CCD) camera, using a 100× (1.6 NA) oil immersion lens kindly provide by the Nikon Imaging Centre, Heidelberg, Germany. Deconvolution was performed using SVI's Huygens Deconvolution Software (http://www.svi.nl) and further processed using ImageJ 1.34r software.

### Immunoblot Analysis

For immunoblot analysis intracellular parasites were cultivated in HFF cells in absence or presence of 0.1–1 µM Shld-1 for 10 h. Subsequently parasites were harvested and washed twiced in ice-cold PBS and lysed in 40 µl ice-cold RIPA buffer (150 mM NaCl, 1% Triton X-100, 0.5% Deoxycholat, 0.1% SDS, 50 mM Tris pH8.0 and 1 mM EDTA). SDS page and western blot analysis was performed as described previously [40]. Per experiment 5×10^6^ parasites/lane were loaded on 12% SDS – polyacrylamid gel using reducing conditions with 100 mM DTT. For detection, polyclonal anti-FKBP12 (1∶500, Affinity BioReagents) was used and a polyclonal anti-Mic8Nt (1∶1000, [46]) as internal control.

### Growth Assay

The growth assay was performed as previously described [47]. Monolayer of HFF, grown in six-well plates, were infected with 500 parasites per well in absence or presence of different Shld-1 concentrations. After 7 days of incubation at normal growth conditions (37°C, 5% CO_2_), cells were fixed 10 min with −20°C methanol 100%, stained for 10 min with Giemsa stain and washed with PBS. Images were captured with a 10× (0.07 NA) objective on a Leica DMIL microscope equipped with a Leica DFC320 camera. Images were further processed with Photoshop (Adobe Systems Inc., USA).

### Replication Assay

For the analysis of the intracellular growth 3×10^4^ and 3×10^5^ freshly released extracellular parasites were pre-incubated in absence and presence of 1 µM Shld-1 for 1 h. Subsequently, parasites were allowed to invade HFF cells for 4 h. After removing non invaded parasites by washing with PBS, parasites were incubated in absence and presences of 1 µM Shld-1 for 24 h. To determine the replication efficiency an immunofluorescence analysis was performed and the number of nuclei per vacuoles was counted.

### Electron Microscopy

Monolayer of HFF, grown on 6 cm dishes, were infected with ddRab11B_N125I_ parasites and cultured for 24 and 36 h in absence or presence of 1 µM Shld-1 and subsequently fixed with 2.5% glutaraldehyde in 0.1 M phosphate buffer pH 7.4. Samples were processed for routine electron microscopy as described previously [48]. In summary samples were post-fixed in osmium tetroxide, dehydrated, treated with propylene oxide and embedded in Spurr's epoxy resin. Thin sections were stained with uranyl acetate and lead citrated prior to examination in a JEOL 1200EX electron microscope.

### Multiple Sequence Alignment and Phylogenetic Analysis

Protein sequences were retrieved from NCBI/GenBank, ToxoDB.org, PlasmoDB.org, and the *Cyanidioschyzon merolae* database (http://merolae.biol.s.u-tokyo.ac.jp/). After randomizing sequence order, alignments were created with ClustalX v1.83 [49] using the default Pairwise and Multiple Alignment Parameters (the sequence Alignment can be downloaded as supplemental data). The variable C-terminal region (after amino acid position 187 in TgRab11B, accession XP_002369905) of the alignment was removed before carrying out phylogenetic analysis using the PHYLIP v3.69 programs SEQBOOT, PROML, PROTDIST, NEIGHBOR, and CONSENSE [50]. PROTDIST and PROML were run employing the Jones–Taylor–Thornton amino acid substitution matrix, and gamma distribution parameters for four variable and one unvariable rate categories were estimated with TREEPUZZLE v5.2 [51]. Sequence input order was randomized in PROML and NEIGHBOR, and global rearrangements were carried out in PROML. For bootstrapping, 100 re-sampled replicates were generated with SEQBOOT and an unrooted Majority Rule consensus tree was created with CONSENSE. Phylogenetic trees were visualized with TREEVIEW v1.6.6 [52].

## Supporting Information

Data S1 Alignment used to produce phylogenetic tree shown in Figure 1
(0.05 MB DOC)

Figure S1 Alignment of Rab11 proteins. ClustalW alignment of indicated Rab11-GTPases. Highly conserved regions are indicated in red. Alveolate specific amino acid substitutions in Rab11B can be identified in critical regions, such as the P-loop, the Switch I and Switch II region (indicated by black arrows). Note that in case of some alveolates duplications of Rab11B occurred (i.e. PtRab11-1 and PtRab11-2). (*Hs: Homo sapiens*; *Sc: S.cerevisia*, *Tth: Tetrahymena thermophila*; *Pt: Paramecium tetraurelia*; *Pf:P.falciparum*; *Cp: Cryptosporidium parvum*; *Cm: Cyanidioschyzon merolae*; *Cr: Chlamydomonas reinhardtii*; *Tb: Trypanosoma brucei*; *Gl:Giardia lamblia*; *Dd: Dictyostelium discoidum*; *Nt: Nicotiana tabacum*; *Pm: Perkinsus marinus*; *Tg: Toxoplasma gondii*; *Bb: Babesia bovis*).
(7.86 MB TIF)

Figure S2 Quantification of replication of the dominant negative Rab11B parasites. (A) Time course of ddRab11BN125I parasites grown in absence and presence of 1 µM Shld-1 over 24 h, 36 h and 48 h. Scale bars represent 10 µm. Parasites were double-labelled with anti-myc (green) and anti-IMC1 (red) to visualise Rab11B and IMC1. Note that IMC formation is completely blocked after 24 hours, resulting in multi-nucleated parasites. (B) Quantification of nuclear division of the same parasite strain treated with or without Shld-1 (−/− parasites not treated with Shld-1; −/+ parasites treated with Shld1 after invasion and +/+, parasites treated with Shld1 before and after invasion). Number of nuclei per parasitophorous vacuole (PV) was determined. Mean values of independent experiments s.d. are shown. In presence of Shld-1 expression of ddRab11BN125I results in a decrease in replication rate and a tendency to asynchronised nuclear division, as indicated by an increased number of odd nuclei per PV. Asterisks indicate significant differences in asynchronized replication (*P*<0.05, two tailed Student's t-test). (C) Quantification of the observed defect in IMC formation of the same parasites (−/− parasites not treated with Shld-1; −/+ parasites treated with Shld1 after invasion and +/+, parasites treated with Shld1 before and after invasion). Total number of parasitophorous vacuoles was counted showing a deformed IMC. Parasites treated with 1 µM Shld-1 showed a defect in IMC formation (100%).
(4.02 MB TIF)

Table S1 List of protein sequences within orthology group OG4_21991
(0.04 MB DOC)
