# Supplementary material for: Biogenesis of the Inner Membrane Complex Is Dependent on Vesicular Transport by the Alveolate Specific GTPase Rab11B
Source: PLoS Pathog. 2010 Jul 29;6(7):e1001029. doi: 10.1371/journal.ppat.1001029 (PMC2912401; doi:10.1371/journal.ppat.1001029)
Supplement: Data S1 — Alignment used to produce phylogenetic tree shown in Figure 1 (0.05 MB DOC) [file ppat.1001029.s001.doc]

Alignment: D:\Work\Paper\Rab11B\JCB\AlignRab11.txt

....|....| ....|....| ....|....| ....|....| ....|....| ....|....|

5 15 25 35 45 55

_HsRab2_NP ---YAYLFKY IIIGDTGVGK SCLLLQFTDK RFQPVHDLTI GVEFGARMIT I---DGKQIK

_DmRab2_NP ---YAYLFKY IIIGDTGVGK SCLLLQFTDK RFQPVHDLTI GVEFGARMIT I---DGKQIK

_CrRab2_XP ---YAYLFKY IIIGDTGVGK SCLLLQFTDK RFQPVHDLTI GVEFGARMIN I---DGKQIK

_AtRab2_CA ---YAYLFKY IIIGDTGVGK SCLLLQFTDK RFQPVHDLTI GVEFGARMIT I---DNKPIK

_PiRab2_EE ---YAYLFKY IIIGDTGVGK SCLLLQFTDK RFQPVHDLTI GVEFGARMIN I---ENKQIK

_TgRab2_EE ---YQYLFKY IIIGDTGVGK SCLLLQFTDK RFRTDHDLTI GVEFGARLVS I---AGRQVK

_PfRab2_PF ---YEYLFKY IIIGDTGVGK SCLLLQFTDK RFRADHDLTI GVEFGARLIN L---DNKQIK

_TcRab2_XP ---HHYVFKY IIIGDSGVGK SCLLLQFTDK RFEPLHDLTI GVEFGARVVT I---QQKNVK

_NcRab8_XP NRNYDFLIKL LLIGDSGVGK SCCLLRFSED SFTPSFITTI GIDFKIRTIE L---DGKRVK

_CnRab8_XP GQHYDFLIKL LLIGDSGVGK SCLLLRFCED SWTPSFITTI GIDFKIRTIE L---DGKRIK

_AtRab8_NP RADYDYLIKL LLIGDSGVGK SCLLLRFSDG SFTTSFITTI GIDFKIRTIE L---DGKRIK

_PiRab8_EE -SKYDLLIKL LLIGDSGVGK SCVLLRYSDD SFTTSFITTI GIDFKVKTID V---DGKRIK

_HsRab8_NP -KTYDYLFKL LLIGDSGVGK TCLLFRFSED AFNTTFISTI GIDFKIRTIE L---DGKKIK

_DmRab8_NP -KTYDYLFKL LLIGDSGVGK TCILFRFSED AFNTTFISTI GIDFKIRTIE L---DNKKIK

_TgRab1_XP ---LDHLFKL VLIGDSGVGK SCLLLRFSDD AFTESYITTI GVDFRFRTIN V---DNEIVK

_PiRab1_EE -RDYDHLFKL VLIGDSGVGK SCLLLRFADD AFTESYITTI GVDFRFRTVK I---DNKTVK

_PfRab1_PF -RDYDYLYKI ILIGDSGVGK SCILLRFSDD HFTESYITTI GVDFRFRTIK V---DDKIVK

_PmRab1_EE -RDYDHLFKL VLIGDSGVGK SCLLLRFADD SFTESYITTI GVDFRFRTIP V---SDKTVK

_TgRab1_XP -PEYDYLFKL LLIGDSGVGK SCLLLRFADD TYTESYISTI GVDFKIRTID L---DGKTVK

_PmRab1_EE -PEYDYLFKL LLIGDSGVGK SCLLLRFADD TYTESYISTI GVDFKIRTIE L---DGKTVK

_ChRab1_EA -PEYDYLFKL LLIGDSGVGK SCLLLRFADD TYTDSYISTI GVDFKIRTIS L---ENKTVK

_HsRab1_NP -PEYDYLFKL LLIGDSGVGK SCLLLRFADD TYTESYISTI GVDFKIRTIE L---DGKTIK

_UmRab1_XP -ADYDYLFKL LLIGDSGVGK SCLLLRFADD TYTESYISTI GVDFKIRTIE L---EGKTVK

_TcRab1_XP -AEYDHLFKL LLIGDSGVGK SCLLLRFADD SYTESYISTI GVDFKIRTLN L---DGKVVK

_AtRab1_NP -NEYDYLFKL LLIGDSSVGK SCLLLRFADD AYIDSYISTI GVDFKIRTIE Q---DGKTIK

CpRab11B_E PDEYDHLYKI ILVGDATVGK THLLSRYTRD ALPKTPQPTI GVEFATRTVP LS--IGGTVK

TgRab11B_X SEDYDHLYKV ILVGDATVGK THLLSRYIRG TLPKSPKATI GVEFATRTVP LA--VGGTVK

PfRab11B_C -EEYDHLYKI ILVGDATVGK THLLSRYIRG SLPSVAKATI GVEFATRTIP LA--VGGTVK

PmRab11B_E DDEYDHLYKI VLVGDATVGK THLLSRYIKG TLPRAPTATI GVEFATRTVP LA--VGGTVK

TpRab11B_E DEDYEHVYKI ILLGDATVGK SHLLCRYIRG DLPVQAKATI GVEFATRTVP LA--SGGSVK

BbRab11B_X QDDYDNVYKI ILLGDATVGK SHLLSHYIRG TLPKQAKATI GVEFATRTVP LA--SGGTVK

PtRab11-1_ -EDYSDFFKV VLVGDAGVGK THLMTRYVKG CLPKNAVPTI GIEFAGKTVT LQ--NGKKVK

TthRab11_X -ATY----KI VLVGDTSVGK THLLKRYSKN ELPKNSAPTI GVEFGTKEVT LK--DGTKVK

PmRab11_XP KSNSYIQLQV VLLGDAGVGK TYILNQYVRG QQPRNNLPTI GIEFATKTVT LQ--DGGKIQ

TthRab11_X SETHTHMYKI VLVGDAGVGK THIINRYVKG QLPHAIIPTI GIEFATKTVT LR--DGGTIK

CpRab11A_X DEHYDYLYKI VLIGDSGVGK SNLLSRFTRD EFNLESKSTI GVEFATKSII T---EGKVIK

TgRab11A_X DEYYDYLYKI VLIGDSGVGK SNMLSRFTRD EFNLESKSTI GVEFATKSVY LD--EGKVIK

PfRab11A_C EDYYDYLFKI VLIGDSGVGK SNLLSRFTRD EFNLESKSTI GVEFATKSIQ LK--NNKIIK

PsiRab11A_ DDEYDYLFKV VLIGDSGVGK SNLLSRFTRN EFCLESKSTI GVEFATRTVQ V---EGKTIK

AthRab11C_ DEEYDYLFKV VLIGDSGVGK SNLLSRFTRN EFCLESKSTI GVEFATRTLQ V---EGRTVK

AthRab11A_ DQEYDYLFKI VLIGDSGVGK SNILSRFTRN EFCLESKSTI GVEFATRTTQ V---EGKTIK

AthRab11B_ DDDYDYLFKL VLIGDSGVGK SNLLSRFTRN EFSIESKSTI GVEFATRSVH V---DEKIIK

PsiRab11B_ DDDYDYLFKV VLIGDSGVGK SNLLSRFTRN EFSLESKSTI GVEFATRSIN V---DGKMIK

CrRab11_XP DDDYDYLFKV VLIGDSGVGK SNLLSRFTRN EFSLESKSTI GVEFATRSIQ V---DGKTIK

PtriRab11_ DEEYDYLFKI VLIGDSGVGK SNLLSRFTRN EFNLESKSTI GVEFATKSIQ A---EGKTIK

TpsRab11A1 DEEYDYLFKV VLIGDSGVGK SNLLSRFTRN EFNLESKSTI GVEFATKSIQ T---EGKTIK

PiRab11A_E EDEYDYLFKI VLIGDSGVGK SNLLSRFTRN EFNLESKSTI GVEFATKSIV A---EGKTIK

NcRab11_CA -DEYDFLFKV VLIGDSGVGK SNLLSRFTRN EFNLDSKSTI GVEFATRSIQ V---DSKTIK

AflaRab11_ -DEYDFLFKV VLIGDSGVGK SNLLSRFTRN EFNLDSKSTI GVEFATRSIQ V---DSKTIK

UmRab11_XP -SNYDYLFKV VLIGDSGTGK SNLLSRFTRN EFSLESKSTI GVEFATRSIS V---DGKTVK

HsRab11A_C DDEYDYLFKV VLIGDSGVGK SNLLSRFTRN EFNLESKSTI GVEFATRSIQ V---DGKTIK

DrRab11A_N DDEYDYLFKV VLIGDSGVGK SNLLSRFTRN EFNLESKSTI GVEFATRSIQ V---DGKTVK

HsRab11B_A DDEYDYLFKV VLIGDSGVGK SNLLSRFTRN EFNLESKSTI GVEFATRSIQ V---DGKTIK

DrRab11B_N DDEYDYLFRV VLIGDSGVGK SNLLSRFTRN EFNLESKSTI GVEFATRSIQ V---DGKTIK

DmRab11_NP EDEYDYLFKV VLIGDSGVGK SNLLSRFTRN EFNLESKSTI GVEFATRSIE V---DGKTIK

TrichRab11 DDEYDYLFKV VLIGDSGVGK SNLLSRFTRN EFNLESKSTI GVEFATRSIQ V---DGKTIK

MbRab11_XP DDEYDFLFKI VLIGDSGVGK SNLLSRFTRN EFNLESKSTI GVEFATRSIK V---ENKTIK

DdRab11_XP QEEYDYLYKI VLIGDSGVGK SNLLSRFTRN EFSLETKSTI GVEFATRTIQ T---EGKTIK

GraRab11A1 VRPVDYLFKV VLIGDAGVGK SNLLSRFTRN EFSLESKSTI GVEFATRSIQ C---EGKIIK

CmRab11_CM DDEYDYLFKI VLIGDSGVGK SNLLSRFTRN EFNLESKSTI GVEFATRSVQ T---DGKVIK

SpRab11_NP EDEYDYLFKT VLIGDSGVGK SNLLMRFTRN EFNIESKSTI GVEFATRNIV L---DNKKIK

PmRab11A_E EEDYDYLFKV VLIGDSGVGK SNILSRFTRG EFNLESRSTI GVEFATKSVR I---GSKVVK

PmRab11_EE REDYDYLFKV VLIGDSGVGK SNILSRFTRG EFNLESRSTI GVEFATKSVR I---GEKVVK

ScRab11_P3 GYDYDLLFKI VLIGDSGVGK SNLLSRFTKN EFNMDSKSTI GVEFATRTLE I---DGKRIK

TvRab11_AA --EPDFLLKI VLIGDSGVGK TNLLSRFARD QFNPDSKSTI GVEFATKTLE I---EGKTVK

TvRab11_XP --EPDYLLKI VLIGDSGVGK TNLLARFTRD QFNPESKSTI GVEFASKTMQ I---EGKTIK

TthRab11_0 EDEYDFLFKI VLIGDSGVGK TNLLSRFTKN EFNMDSKPTI GVEFATKTVI T---ENKYIK

EhRab11A_B QEDYDFLYKI VLIGESGVGK SNLLLRFTRN EFDAEKRSTI GVEFATRSIQ H---EGKVIR

EhRab11B_X SEEYDFLYKI VLVGESGVGK SNLLLRFTRN EFDPDKRSTI GVEFATRSIN Y---CDKNIR

BbRab11A_X -QSYDYLFKI VLIGDSNVGK SNLLDRFVKG NFKLDSKSTI GVEFATKTVT LN--NGKIAK

TpRab11A_X -QNYDYLFKI VLIGDSNVGK SNLLDRFVKG NFKLDSKSTI GVEFATKNVN LR--NGKVAK

TcRab11_XP MEDTNLTFKV VIIGDSGVGK SNLMTRYTAN EFSQDTPATI GVEFMTKSIK I---EGRDAK

LmRab11_XP MEETNLSFKI VLIGDSGVGK SNLMTRYTTN EFNQETPSTI GVEFMTKSVK I---ESRDAK

PiRab11_EE PPITESILKV VLIGDSGVGK SNLVMRFTKN KYMPHSVQTV GFEFATKTIR V---GDRRLR

_DmRab5_NP -QNKSCQFKL VLLGESAVGK SSLVLRFVKG QFHEYQESTI GAAFLTQTIC I--ED-TVVK

_HsRab5_NP --NKICQFKL VLLGESAVGK SSLVLRFVKG QFHEYQESTI GAAFLTQTVC L--DD-TTVK

_PiRab5_EE --GKTCHFKL VLLGDTAVGK SCLVVRFVRD EFFEFQEPTI GAAFLTQTVG L--EDGLTVK

_AtRab5_NP --NKSINAKL VLLGDVGAGK SSLVLRFVKD QFVEFQESTI GAAFFSQTLA V--ND-ATVK

_CrRab5_ED --AQQQTAKL VLLGEMGSGK SSLVLRYVKG QFFDYQASTV GAAFLTKTLP ----E-LNVK

_TgRab5_AA -LPKTLHFKL VLLGDTSVGK SCLVVRFAKD EFYEYQESTI GAAFMTQSVN ---LGSCIVK

_PfRab5_CA -STKVFNSKL VLLGDTSVGK SCIVVRFAKN EFYEYQESTI GAAFMTQLID ---IGECTIK

_PmRab5_EE -PLTVLHHKL VLLGDASVGK SCMVVRFARG EFYEYQEPTI GAAFMTQTVS PFPDSPVQIK

....|....| ....|....| ....|....| ....|....| ....|....| ....|....|

65 75 85 95 105 115

_HsRab2_NP LQIWDTAGQE SFRSITRSYY RGAAGALLVY DITRRDTFNH LTTWLEDARQ HSN-SNMVIM

_DmRab2_NP LQIWDTAGQE AFRSITRSYY RGAAGALLVY DITRRETFNH LTTWLEDARQ HSN-SNMVIM

_CrRab2_XP LQIWDTAGQE SFRSITRSYY RGAAGALLVY DITRRETFNH LASWLEDARQ HAN-PNMTIM

_AtRab2_CA LQIWDTAGQE SFRSITRSYY RGAAGALLVY DITRRETFNH LASWLEDARQ HAN-ANMTIM

_PiRab2_EE LQIWDTAGQE SFRSITRSYY RGAAGALLVY DITRRETFNH LTRWLEEARQ NSN-SNMAIM

_TgRab2_EE LQIWDTAGQE SFRSITRSYY RGAAGALLVY DITRRDTFLH LTRWLDEVRQ NSN-PHMTIM

_PfRab2_PF LQIWDTAGQE SFRSITRSYY RGAAGALLVY DITRRETFNH LNRWLDEVRQ NSN-PHMAII

_TcRab2_XP LQIWDTAGQE SFRSITRSYY RGACGALLVY DVTRRETFTH LQTWLEDAKA NAN-TAIVIM

_NcRab8_XP LQIWDTAGQE RFRTITTAYY RGAMGILLVY DVTDERSFNN IRTWFANVEQ HAT-EGVNKI

_CnRab8_XP LQIWDTAGQE RFRTITTAYY RGAMGILLVY DVTDEKSFNN IRTWLSNIEQ HAS-PGVNKI

_AtRab8_NP LQIWDTAGQE RFRTITTAYY RGAMGILLVY DVTDESSFNN IRNWIRNIEQ HAS-DNVNKI

_PiRab8_EE LQIWDTAGQE RFRTITTAYY RGAMGILMVY DVTDDHSFQN IRNWMTQIRQ NAS-SNVNKI

_HsRab8_NP LQIWDTAGQE RFRTITTAYY RGAMGIMLVY DITNEKSFDN IKNWIRNIEE HAS-SDVERM

_DmRab8_NP LQIWDTAGQE RFRTITTAYY RGAMGIMLVY DITQEKSFEN IKNWIRNIEE NAS-ADVEKM

_TgRab1_XP LQIWDTAGQE RFRTITSAYY RGADGIVLVY DVTDRESFLH VDEWLAEVNR YAN-ENTCKI

_PiRab1_EE LQIWDTAGQE RFRTITSAYY RGADGIIMVY DVTSQESFDH VNDWLNEVNR YAS-EGTCKL

_PfRab1_PF LQIWDTAGQE RFRTITSAYY RGADGIIIIY DTTDRNSFLH INDWMNEINK YTN-EDTCKL

_PmRab1_EE LQIWDTAGQE RFRTITSAYY RGADGIMMVY DCTHRESFEN IDNWLSEVNR YAN-DSTVKI

_TgRab1_XP LQIWDTAGQE RFRTITSSYY RGAHGIIIVY DVTDRESFNN VKNWMMEIDK YAM-EGVSKL

_PmRab1_EE LQIWDTAGQE RFRTITSSYY RGAHGIIIVY DVTDRESFNN VKHWVQEIDK YAT-ENVSKL

_ChRab1_EA LQIWDTAGQE RFRTITSSYY RGAHGIIIVY DVTDRDSFDN VKQWIQEIDR YAM-ENVNKL

_HsRab1_NP LQIWDTAGQE RFRTITSSYY RGAHGIIVVY DVTDQESYAN VKQWLQEIDR YAS-ENVNKL

_UmRab1_XP LQIWDTAGQE RFRTITSSYY RGAHGIIVVY DVTDNDTFSN VKQWLQEIDR YAC-EGVNKL

_TcRab1_XP LQIWDTAGQE RFRTITSSYY RGAHGIIIVY DTTDMESFNN VKTWLSEIDK FAS-ENVNKL

_AtRab1_NP LQIWDTAGQE RFRTITSSYY RGAHGIIIVY DCTEMESFNN VKQWLSEIDR YAN-ESVCKL

CpRab11B_E AQIWDTAGQE RYRAITRAHY RRSVGALLVY DITRKSSFLN ASKWLEDIKQ NSE-PDIVVM

TgRab11B_X AQIWDTAGQE RYRSITSAHY RRAVGALLVY DVTRKSTFLN ASKWLEELRQ NSE-PDIVIM

PfRab11B_C AQIWDTAGQE RYRSITSAHY RRSAGAILVY DITKKKTFLS ISKWLEEIRQ NAD-KDIVIM

PmRab11B_E AQIWDTAGQE RYRAITSAHY RRAVGALLVY DVTRRNTFLN CSKWMEELRQ NAE-PDIVIL

TpRab11B_E AQIWDTAGQE RYRSITSAHY RRAVGALLVY DVTNRSSFYN CKKWLDELRA SSY-DDIVIL

BbRab11B_X AQIWDTAGQE RYRSITSAHY RRAVGALLVY DITNRQSFYN CHKWLDELRM AAE-PDIVVV

PtRab11-1_ AQIWDTAGQE RYRGITSTHF RKAGGALVVY DVTKEKTFES VVKWMEDLRY QAE-PDVVIM

TthRab11_X AQIWDTAGQE RYRGILSLHF RRAVGALLVY DITKEKTYNS IMKWMEDLKY LAD-PDIVIM

PmRab11_XP VQIWDTAGQE RYRAITTNHF RGAGGALLVY DITKEKTFEN LTRWMEELKT AAN-KDVVMY

TthRab11_X TQIWDTAGQE KYRSITSAHY RKAVGALLVY DVTKEKSFES IQRWLEEIRL HAD-KDIVLM

CpRab11A_X AQIWDTAGQE RYRAITSAYY RGAVGALLVY DISKRSSFEN VERWLKELRD HAD-PNIVVL

TgRab11A_X AQIWDTAGQE RYRAITSAYY RGAVGALLVY DITKRQSFEN VERWLKELRD HAD-PNIVIL

PfRab11A_C AQIWDTAGQE RYRAITSAYY RGAVGALLVY DITKKNSFEN IEKWLKELRD NAD-SNIVIL

PsiRab11A_ AQIWDTAGQE RYRAITSAYY RGAVGALLVY DITKPTTFEN VGRWLKELRD HAD-SNIVIM

AthRab11C_ AQIWDTAGQE RYRAITSAYY RGALGALLVY DVTKPTTFEN VSRWLKELRD HAD-SNIVIM

AthRab11A_ AQIWDTAGQE RYRAITSAYY RGAVGALLVY DITKRQTFDN VLRWLRELRD HAD-SNIVIM

AthRab11B_ AQLWDTAGQE RYRAITSAYY RGAVGALLVY DITRHITFEN VERWLKELRD HTD-ANVVIM

PsiRab11B_ AQIWDTAGQE RYRAITSAYY RGAVGALLVY DITRHVTFEN VERWLKELRD HTE-HNIVVM

CrRab11_XP AQIWDTAGQE RYRAITSAYY RGAVGALLVY DITKSVTFEN VERWLKELRD HAD-SNIVIM

PtriRab11_ AQIWDTAGQE RYRAITSAYY RGAVGALLVY DISKHGTFEN VERWLKELRD HAE-ANIVVM

TpsRab11A1 AQIWDTAGQE RYRAITSAYY RGAVGALLVY DISKHGTFEN VERWLKELRD HAE-ANIVVM

PiRab11A_E AQIWDTAGQE RYRAITSAYY RGAVGALLVY DITKHGTFEN VERWLKELRD HAD-ANTVIM

NcRab11_CA AQIWDTAGQE RYRAITSAYY RGAVGALLVY DISKGVTFEN VNRWLKELRD HAD-QNIVIM

AflaRab11_ AQIWDTAGQE RYRAITSAYY RGAVGALLVY DISKHQTYDN VNRWLKELRD HAD-SNIVIM

UmRab11_XP AQIWDTAGQE RYRAITSAYY RGAVGALLVY DIAKHPTYVN VSRWLKELRD HAD-SNIVIM

HsRab11A_C AQIWDTAGQE RYRAITSAYY RGAVGALLVY DIAKHLTYEN VERWLKELRD HAD-SNIVIM

DrRab11A_N AQIWDTAGQE RYRAITSAYY RGAVGALLVY DIAKHLTYEN VERWLKELRD HAD-SNIVIM

HsRab11B_A AQIWDTAGQE RYRAITSAYY RGAVGALLVY DIAKHLTYEN VERWLKELRD HAD-SDIVIM

DrRab11B_N AQIWDTAGQE RYRAITSAYY RGAVGALLVY DIAKHLTYEN VERWLKELRD HAD-NNIVIM

DmRab11_NP AQIWDTAGQE RYRAITSAYY RGAVGALLVY DIAKHLTYEN VERWLRELRD HAD-QNIVIM

TrichRab11 AQIWDTAGQE RYRAITSAYY RGAVGALLVY DIAKHLTYEN IERWLKELRD HAD-ANIVIM

MbRab11_XP AQIWDTAGQE RYRAITSAYY RGAVGALLVY DIAKHLTYEN VERWLKELRD HAD-SNIVIM

DdRab11_XP AQVWDTAGQE RYRAITSAYY RGAVGALLVY DIAKQATYKS VERWILELRE NAD-RNIEIM

GraRab11A1 AQIWDTAGQE RYRAITSAYY RGAVGALLVY DITKKDSFDN VQRWLKELKD HAD-ANIVIM

CmRab11_CM AQIWDTAGQE RYRAITSAYY RGAVGALLVY DISKRSSFEN AERWLKELRD HAD-QNIVIM

SpRab11_NP AQIWDTAGQE RYRAITSAYY RGAVGALIVY DITKQSSFDN VGRWLKELRE HAD-SNIVIM

PmRab11A_E AQIWDTAGQE RYRAITSAYY RGAVGALLVY DISKRSSFEN AARWLKELRE HAD-SDIVVM

PmRab11_EE AQIWDTAGQE RYRAITSAYY RGAVGALLVY DISKRSSFDN AARWLKELRE HAD-SDIVVM

ScRab11_P3 AQIWDTAGQE RYRAITSAYY RGAVGALIVY DISKSSSYEN CNHWLSELRE NAD-DNVAVG

TvRab11_AA AQIWDTAGQE RYRAITSAYY RGAIGALLLY DITASLTFNS LSRWLQELRE NAD-SNIVVM

TvRab11_XP AQIWDTAGQE RYRAITSAYY RGAIGALLLY DITASLTFNS LEKWLKELRE NAD-EKIIVM

TthRab11_0 AQIWDTAGQE KYRAITNAYY RGAVGALTLY DITKQQTFDN VKKWLHELRE YAD-ANIICM

EhRab11A_B AQIWDTAGQE RYRAITNAYY RGAVGALVVY DITKENTFKS IERWLSELKD NAD-PKIVIM

EhRab11B_X AQIWDTAGQE RYRAITNAYY RGALGALVVY DITKKTSFES VEKWLAELHE NAD-KKVVQM

BbRab11A_X AQIWDTAGQE RYRAITSAYY RGAMGAIIVY DIACKTSFTN VSKWLTELHD YAD-SNITIC

TpRab11A_X AQIWDTAGQE RYRAITSAYY RGARGAIVVY DIASKQSFYN VSRWLSELNE YGD-ANMIIA

TcRab11_XP VQIWDTAGQE RFRAISRSIY HGAKGAMLVY DITNQTSFDS IPTWLQELRV FVP-ATCSIF

LmRab11_XP IQIWDTAGQE RFRAISRSIY HGAKGAMLVY DITNQTSFDS IPTWLQELRV FVP-ATCCIF

PiRab11_EE AQIWDTAGQE RFQSLTAAYY RNAVGAMIVY DITNRSSFEH VTGWLAQVHE HSH-ESLVLI

_DmRab5_NP FEIWDTAGQE RYHSLAPMYY RGAQAAIVVY DIQNQDSFQR AKTWVKELHK QAS-PNIVIA

_HsRab5_NP FEIWDTAGQE RYHSLAPMYY RGAQAAIVVY DITNTDTFAR AKNWVKELQR QAS-PNIVIA

_PiRab5_EE FEIWDTAGQG ---NLAPMYY RGAAAAIVVY DVTNKDSFTG AKSWVKELQR RGD-PNVVIA

_AtRab5_NP FEIWDTAGQE RYHSLAPMYY RGAAAAIIVF DVTNQASFER AKKWVQELQA QGN-PNMVMA

_CrRab5_ED FEIWDTAGQE RYHSLAPMYY RGAAAAIIVY DITSPDSFTR AKSWVRELQR QGN-PNMIMA

_TgRab5_AA FEIWDTAGQE RYRSLAPMYY RGAAAAVVVY DISNRDSFQG AKSWVQELQS VNDRSNVVIA

_PfRab5_CA FEIWDTAGQE RYRSLAPMYY RGASAAVIVY DITNKKSFEG AKGWIHELKS VHS-NDIIIA

_PmRab5_EE FEIWDTAGQE RYRSLAPMYY RGAAAAVVVY DITSRESFEG AKRWVNELRS SHN-PDVVIA

....|....| ....|....| ....|....| ....|....| ....|....| ....|....|

125 135 145 155 165 175

_HsRab2_NP LIGNKSDLES ----RREVKK EEGEAFAREH GLI-FMETSA KTASNVEEAF INTAKEIYEK

_DmRab2_NP LIGNKSDLDS ----RREVKK EEGEAFAREH GLV-FMETSA RTAANVEEAF INTAKEIYEK

_CrRab2_XP LIGNKCDLTH ----RRAVTT EEGEQFAKEH GLI-FLETSA RTAHNVEEAF INTAKEIYKK

_AtRab2_CA LIGNKCDLAH ----RRAVST EEGEQFAKEH GLI-FMEASA KTAQNVEEAF IKTAATIYKK

_PiRab2_EE LIGNKSDLEH ----RRAVSF KEGEQFAKEN GLI-FLETSA KTAANVEDAF VKTASKIYSN

_TgRab2_EE LIGNKSDLE- ----RREVSF DEGAAFARQH GLI-FLETSA KTAQNVDEAF ILTARKIYEN

_PfRab2_PF LVGNKCDLE- ----RREVSA EEGAQFARQN GLI-FLETSA KTAKNVEEAF LYTARKIYDN

_TcRab2_XP LIGNKCDLEA ----KRQVSR EEGESFAKKN NLV-FMETSA KTAQNVDDAF MKTAMMIYEN

_NcRab8_XP LIGNKCDWEE ----KRAVSK EQGQALADEL GIP-FLEVSA KANINIEEAF FSLANDIKKR

_CnRab8_XP LIGNKCDWEE ----KRSVTI EQGRALADEF GLR-FLETSA KANEGVEEAF FTLARDIKTR

_AtRab8_NP LVGNKADMDE S---KRAVPT AKGQALADEY GIK-FFETSA KTNLNVEEVF FSIGRDIKQR

_PiRab8_EE LIGNKCDVDP S---ERAVTT KQGQDLADEF GIK-FFETSA KSNENIDEAF RSIAVDIQKR

_HsRab8_NP ILGNKCDMND ----KRQVSK ERGEKLAIDY GIK-FLETSA KSSANVEEAF FTLARDIMTK

_DmRab8_NP LLGNKCELTD ----KRQVSK ERGEQLAIEY GIK-FMETSA KASINVEEAF LTLASDIKAK

_TgRab1_XP LVGNKCEKAD ----DRQVSV EEGQRKAEEL GIS-FIETSA KNAINVDEAF TVVARELIKM

_PiRab1_EE LVGNKSDISD ----NKVVSY ETAKAFADSL SIP-FLETSA KNAQNVEEAF LTMASELITI

_PfRab1_PF LVGNKADCKD ----DIEITT MEGQNKAKEL NIS-FIETSA KDATNVELAF TMITQELIKK

_PmRab1_EE LIGNKSDLKE ----DQQVTP EEGEQKAKAL GFSGFILTSA KDSSNVEKAF SMVSQSLIDT

_TgRab1_XP LVGNKCDLTS ----KRTVTY EEGKEFADSC NMR-FIETSA KNAHNVEQAF HIMASEIKA-

_PmRab1_EE LVGNKTDLTS ----KKVVTY DEGKELADQL GVP-FLETSA KNSHNVEQAF IEMSSEIKS-

_ChRab1_EA LVGNKCDLVS ----KRVVTS DEGRELADSH GIK-FIETSA KNAYNVEQAF HTMAGEIKK-

_HsRab1_NP LVGNKSDLTT ----KKVVDN TTAKEFADSL GIP-FLETSA KNATNVEQAF MTMAAEIKK-

_UmRab1_XP LVGNKSDLTN ----KKVVEY ATAKDFADQL QIP-FLETSA KSATNVEQAF LTMAKQIKD-

_TcRab1_XP LVGNKCDLVL ----KKAVDT QMAKDFADNL GIP-FLETSA KEASNVEEAF TRMAMDIKK-

_AtRab1_NP LIGNKNDMVE ----SKVVST ETGRALADEL GIP-FLETSA KDSINVEQAF LTIAGEIKK-

CpRab11B_E LVGNKLDLVE KDPSKREVPF DIAANFAQEN NLF-FSEASA VTRCNVKHIF EHLLQEVYNQ

TgRab11B_X MVGNKLDLVE KDPTARDVPY ELAAKFAQAN GLY-FSEASA VTAFNVKHIF EHLLQEIYNH

PfRab11B_C LVGNKVDLTE EDETKRKVTY EQGANFAREN NLF-FAEASA VSKLNVKHIF ENLLQEIYNN

PmRab11B_E LVGNKIDLVE KDSSTRQVTT EEATVFAKQS GLF-FAEASA VSSVNVKFIF ENLLQEIYNQ

TpRab11B_E LIANKVDLTN TQENVRVVMA VEGMEFASEN NLH-FFEASA VTGYNVKEIF EFLIQQIYNL

BbRab11B_X LVGNKIDLAI QDPSVRQVHR EQATMFANEW NLH-LFEASA VSGYNVKDVF EFLLQEIHNL

PtRab11-1_ LVGNKIDLVE NNGSARKVQK EDAKNLAQQH KVL-FEESSA VTGQNVGQCF DRLLQEMYKI

TthRab11_X LVGNKLDLVT KTQENRKVSI AEAQQFALDN KLI-FKETSA VLGTNIKEVF EQLLQEIYNQ

PmRab11_XP LVGNKTDLVE RQNNSRKVTR EEGQQFAGEN GLF-FEETSA YRGTNIAQCF EKLIEEMYKK

TthRab11_X LVGNKVDLIH KNPEQRKVSK IEATNFAKQN GLL-FEESSA LFDVNINDVF ERLLEEIYDQ

CpRab11A_X LVGNKSDLRN L----RTVTQ EEACAFSERE GMA-CMEASA LNSSNVDEAF HRILSEIYTL

TgRab11A_X LVGNKSDLKH L----RAVSV EEATKFANRE HLA-FIETSA LDATNVEQAF HQILAEIYLL

PfRab11A_C LVGNKSDLKH L----RVIND NDATQYAKKE KLA-FIETSA LEATNVELAF HQLLNEIYNV

PsiRab11A_ LVGNKSDLKH L----RGVST EDAQSFAEKE GLS-FLETSA LEATNVERAF QTILAEIHRI

AthRab11C_ LIGNKTDLKH L----RAVAT EDAQSYAEKE GLS-FIETSA LEALNVEKAF QTILSEVYRI

AthRab11A_ MAGNKSDLNH L----RSVAE EDGQSLAEKE GLS-FLETSA LEATNVEKAF QTILGEIYHI

AthRab11B_ LVGNKADLRH L----RAVPT EEARSFSERE NMF-FMETSA LDATNVEQAF THVLTQIYRV

PsiRab11B_ LVGNKSDLRH L----RAVST EDAQTFAERE GLY-FIETSA LESTNVENAF KQVLTQIYRI

CrRab11_XP LVGNKSDLKH L----RDVQT EVAQAFCERE GLS-FIETSA LESTNVEKAF QQILTEIYHI

PtriRab11_ LVGNKSDLRH L----RAVET DEAMAFSEQH NLA-FIETSA LDASGVDTAF QRILTEIYRL

TpsRab11A1 LVGNKSDLRH L----RAVET DEAMAFSEQH NLA-FIETSA LDASGVDTAF QRILTEIYRL

PiRab11A_E LVGNKSDLRH L----RAVST EEAMAFAEKN NLA-FIETSA LEATGVDSAF QRILTEIYKL

NcRab11_CA LVGNKSDLRH L----RAVPT EDAKKFAEEN HLS-FIETSA LDATNVELAF QNILTEIYKI

AflaRab11_ LVGNKSDLRH L----RAVPT EEAKQFASEN NLS-FIETSA LDASNVELAF QNILTEIYRI

UmRab11_XP LVGNKSDLRH L----RAVPT EEAKAFAAEN NLS-FIETSA LDASNVEQAF QNILTEIYRI

HsRab11A_C LVGNKSDLRH L----RAVPT DEARAFAEKN GLS-FIETSA LDSTNVEAAF QTILTEIYRI

DrRab11A_N LVGNKSDLRH L----RAVPT DEARAFAEKN GLS-FLETSA LDSTNVETAF QTILTEIYRI

HsRab11B_A LVGNKSDLRH L----RAVPT DEARAFAEKN NLS-FIETSA LDSTNVEEAF KNILTEIYRI

DrRab11B_N LVGNKSDLRH L----RAVPT DEARAFAEKN NLS-FIETSA LDSTNVEEAF KNILTEIYRI

DmRab11_NP LVGNKSDLRH L----RSVPT DEAKLFAERN GLS-FIETSA LDSTNVETAF QNILTEIYRI

TrichRab11 LVGNKCDLRH L----RAVPT DEAKNFAEKN TLS-FIETSA LDSTNVETAF QNILTEIYHI

MbRab11_XP LVGNKSDLKH L----RAVPT EEAQKFAEEN DLS-FIETSA LEAENVDDAF TTILTKIYHI

DdRab11_XP LVGNKSDLRH L----REVST DEAKEFSEKH KLT-FIETSA LDSSNVELAF QNILTQIYHI

GraRab11A1 LVGNKSDLQH L----KAVTT DDASAFAESN GLS-FIETSA LDNSNVEEAF RSILSEIYRL

CmRab11_CM LVGNKSDLKH L----RAVTT EEGQQYADAR GLS-FIETSA LDASHVEEAF VRILTDIYRL

SpRab11_NP LVGNKTDLLH L----RAVST EEAQAFAAEN NLS-FIETSA MDASNVEEAF QTVLTEIFRI

PmRab11A_E LVGNKCDLSH L----RAVEE DEAKKFCTDN DLL-FIETSA LEATNVEEAF QEILEDIYKV

PmRab11_EE LVGNKCDLSH L----RAIEE EEAEKFCSDN DLL-FIETSA LEATNVEEAF QNILEDIYKV

ScRab11_P3 LIGNKSDLAH L----RAVPT EESKTFAQEN QLL-FTETSA LNSENVDKAF EELINTIYQK

TvRab11_AA LVGNKSDLQE L----RAVST EDGTGFSQQE NLL-FIETSA RNATNVQEAF TTLITEIVHR

TvRab11_XP LVGNKCDLSE Q----RAVTS ANALEFAKAQ NLL-FIETSA REATNVNEAF QTLICEIVNK

TthRab11_0 LIGNKCDLQE M----RQVKT EDAAKFAEEN NLA-FMETSA AQAINVDQAF QKLITEIYNN

EhRab11A_B IIGNKADLAQ T----REVEE ATAKEFCQKQ NLF-FFETSA LDGTNVETAF KSLLVEIFKQ

EhRab11B_X VIGNKCDLSQ T----REVQT SEGEELAKKN NAF-FFETSA LDGSNVEEAF MTLLKKIYDD

BbRab11A_X LVGNKSDLTH L----REVNK EDGEAFAREN DLL-FFETSC LNSENVDVAF KELLSKIGDK

TpRab11A_X LVGNKSDLTH L----REVTY EDGERYAKSN NLI-FFETSC LSNENIDTTF TELLNLICDN

TcRab11_XP LIGNKCDLEH L----RVVKK EVADRFAREN GLS-FLETSA LEKTNVEKAF EWLAKSVYDL

LmRab11_XP LIGNKCDLEH L----RVVKK EVADRFAREN GLS-FLETSA LERTNVDKAF EWLAKSVYEV

PiRab11_EE LVGNKCDLAH LPES-RQVST LEAARFAAKH SME-FLETSA LDATNVVDAF KKLIVPVGRL

_DmRab5_NP LAGNKADLSN I----RVVEF DEAKQYAEEN GLL-FMETSA KTGMNVNDIF LAIAK-KLPK

_HsRab5_NP LAGNKADLAS K----RAVEF QEAQAYADDN SLL-FMETSA KTAMNVNEIF MAIAK-KLPK

_PiRab5_EE LAGNKADLEA R----RKVEF EEAHQYAEDN DIL-HMETSA KTAVNVKDLF VAIAK-RLPK

_AtRab5_NP LAGNKSDLLD A----RKVTA EDAQTYAQEN GLF-FMETSA KTATNVKEIF YEIAR-RLPR

_CrRab5_ED LAGNKADLEG Q----RAVTV EEAQAYAAEN GLF-YVETSA KTSANVNELF EEIAR-KLPK

_TgRab5_AA LAGNKEDLAA E----RQVPK QEAQQYADEH GIL-FLETSA KTGHNVNELF YEIAA-ALPK

_PfRab5_CA LAGNKNDLEE H----RAVDR ELAESFANSN NIL-FIETSA KTGQNVNELF LRIAK-KLPL

_PmRab5_EE FCGNKSDLAE E----REVPT QRGAEYAQEN DLL-FVETSA KTGANVHHVF VEIGQGELGK

....|..

185

_HsRab2_NP IQEGVFD

_DmRab2_NP IQEGVFD

_CrRab2_XP IQDGVFD

_AtRab2_CA IQDGVFD

_PiRab2_EE IQSGVCD

_TgRab2_EE IQRGIYD

_PfRab2_PF ILEDVYD

_TcRab2_XP VQSGVVD

_NcRab8_XP IIDTSGK

_CnRab8_XP LIDSQPQ

_AtRab8_NP LSDTDSR

_PiRab8_EE LAESEHD

_HsRab8_NP LNRKMND

_DmRab8_NP TEKRMEA

_TgRab1_XP KQSAGAA

_PiRab1_EE REMVGES

_PfRab1_PF KKKKNFT

_PmRab1_EE RAAAAAG

_TgRab1_XP RVQVNQQ

_PmRab1_EE RVKTAPQ

_ChRab1_EA RVQVNSQ

_HsRab1_NP RMGP--G

_UmRab1_XP RMGS--S

_TcRab1_XP RLAAQGG

_AtRab1_NP KMGSQTN

CpRab11B_E KMKDN--

TgRab11B_X RTQGEEF

PfRab11B_C RLKNNNR

PmRab11B_E RSRAQAE

TpRab11B_E KSRLPSV

BbRab11B_X KSRADAT

PtRab11-1_ KSLTPGQ

TthRab11_X KKKNPQP

PmRab11_XP KNLLVTQ

TthRab11_X KCRLQSE

CpRab11A_X RSERQLT

TgRab11A_X RQKKQIE

PfRab11A_C RQKKQAT

PsiRab11A_ ISKKALA

AthRab11C_ ISKKSIS

AthRab11A_ ISKKALA

AthRab11B_ MSRKALD

PsiRab11B_ VSKKALD

CrRab11_XP VSKKVLD

PtriRab11_ MSRRNMQ

TpsRab11A1 MSRRNMA

PiRab11A_E MSRKTIQ

NcRab11_CA VSTKNFD

AflaRab11_ VSSKALD

UmRab11_XP VSNKALQ

HsRab11A_C VSQKQMS

DrRab11A_N VSQKQMS

HsRab11B_A VSQKQIA

DrRab11B_N VSQKQMV

DmRab11_NP VSQKQIR

TrichRab11 VSQKQIK

MbRab11_XP VSQKQVA

DdRab11_XP MSRPSHS

GraRab11A1 GSKKALE

CmRab11_CM VHKRHLA

SpRab11_NP VSNRSLE

PmRab11A_E NSSKPTP

PmRab11_EE NSSKPTP

ScRab11_P3 VSKHQMD

TvRab11_AA LSKQNIV

TvRab11_XP LNKQNVA

TthRab11_0 LKTRPNE

EhRab11A_B -NASAVE

EhRab11B_X -SSKSVD

BbRab11A_X NTMYDNT

TpRab11A_X HEKFGDT

TcRab11_XP VVAPVDA

LmRab11_XP VVTPQTR

PiRab11_EE LSPT---

_DmRab5_NP NDGANNQ

_HsRab5_NP NEPQNAT

_PiRab5_EE NPPQ---

_AtRab5_NP VQPTE-N

_CrRab5_ED PEAAP-R

_TgRab5_AA TRKEHD-

_PfRab5_CA HKKEQER

_PmRab5_EE ANKEEAG
