# Supplementary figures and images for: Biogenesis of the Inner Membrane Complex Is Dependent on Vesicular Transport by the Alveolate Specific GTPase Rab11B
Source: PLoS Pathog. 2010 Jul 29;6(7):e1001029. doi: 10.1371/journal.ppat.1001029 (PMC2912401; doi:10.1371/journal.ppat.1001029)

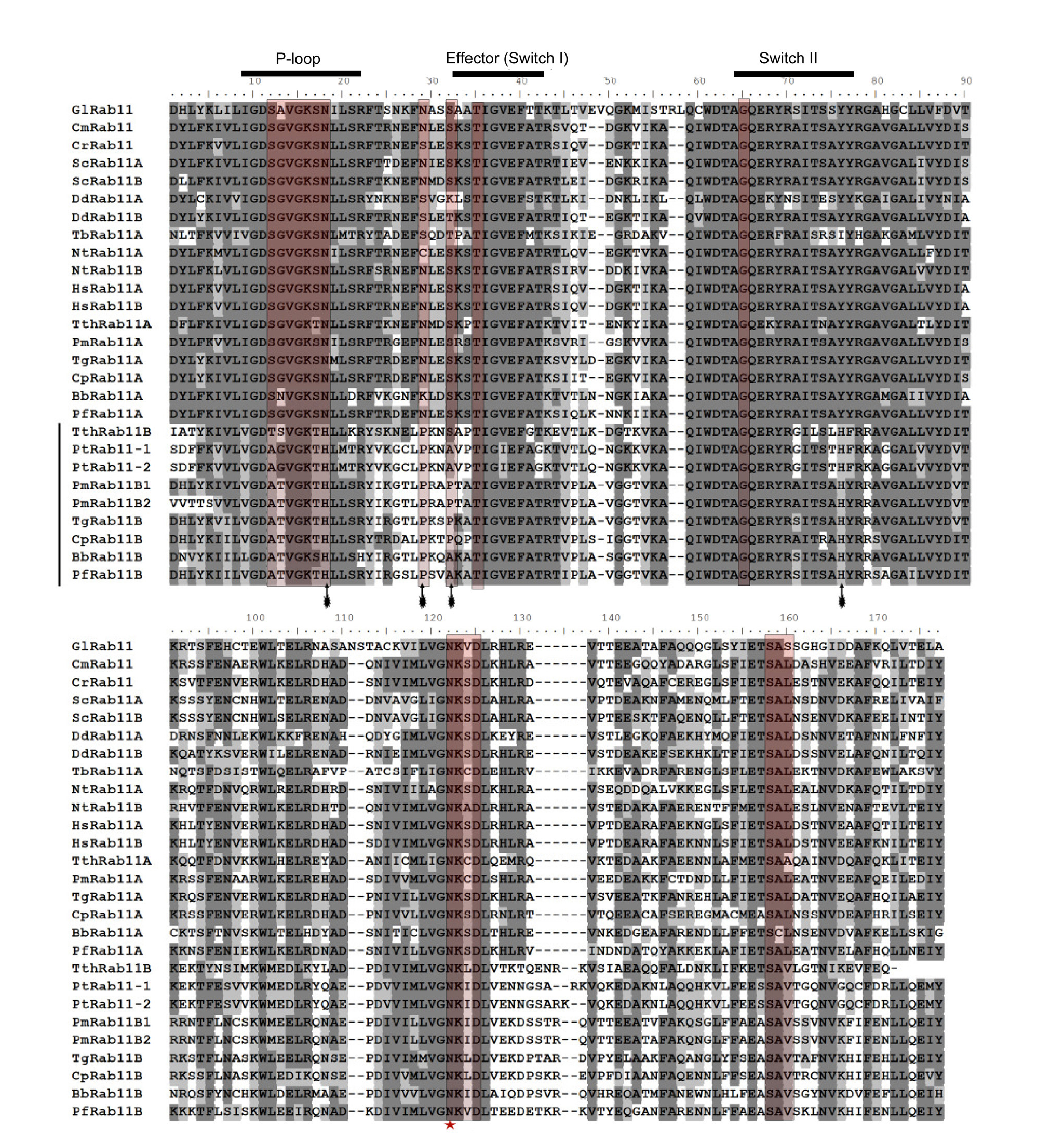

Supplement: Figure S1 — Alignment of Rab11 proteins. ClustalW alignment of indicated Rab11-GTPases. Highly conserved regions are indicated in red. Alveolate specific amino acid substitutions in Rab11B can be identified in critical regions, such as the P-loop, the Switch I and Switch II region (indicated by black arrows). Note that in case of some alveolates duplications of Rab11B occurred (i.e. PtRab11-1 and PtRab11-2). (Hs: Homo sapiens; Sc: S.cerevisia, Tth: Tetrahymena thermophila; Pt: Paramecium tetraurelia; Pf:P.falciparum; Cp: Cryptosporidium parvum; Cm: Cyanidioschyzon merolae; Cr: Chlamydomonas reinhardtii; Tb: Trypanosoma brucei; Gl:Giardia lamblia; Dd: Dictyostelium discoidum; Nt: Nicotiana tabacum; Pm: Perkinsus marinus; Tg: Toxoplasma gondii; Bb: Babesia bovis). (7.86 MB TIF) [file ppat.1001029.s002.tif]

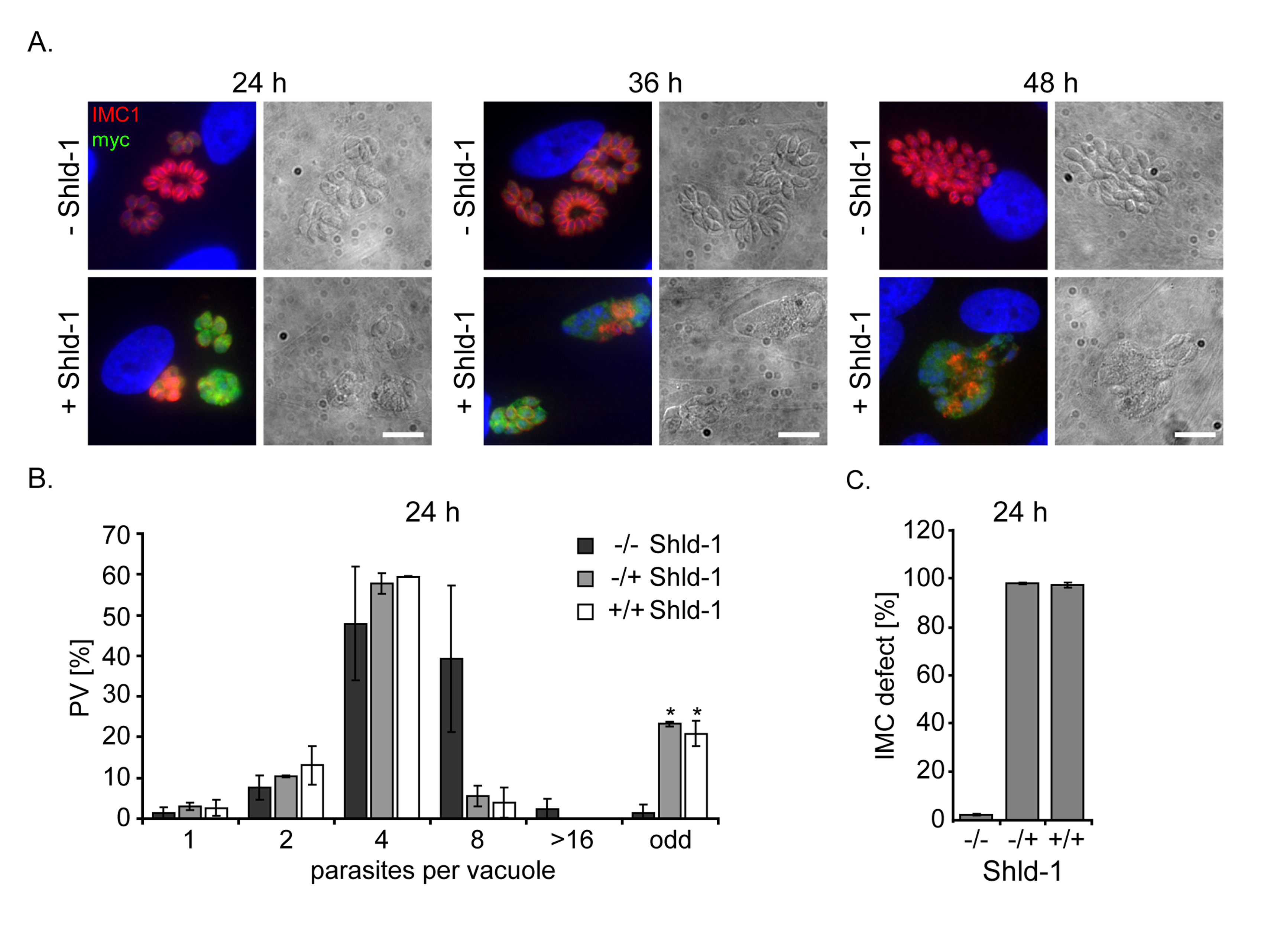

Supplement: Figure S2 — Quantification of replication of the dominant negative Rab11B parasites. (A) Time course of ddRab11BN125I parasites grown in absence and presence of 1 µM Shld-1 over 24 h, 36 h and 48 h. Scale bars represent 10 µm. Parasites were double-labelled with anti-myc (green) and anti-IMC1 (red) to visualise Rab11B and IMC1. Note that IMC formation is completely blocked after 24 hours, resulting in multi-nucleated parasites. (B) Quantification of nuclear division of the same parasite strain treated with or without Shld-1 (−/− parasites not treated with Shld-1; −/+ parasites treated with Shld1 after invasion and +/+, parasites treated with Shld1 before and after invasion). Number of nuclei per parasitophorous vacuole (PV) was determined. Mean values of independent experiments s.d. are shown. In presence of Shld-1 expression of ddRab11BN125I results in a decrease in replication rate and a tendency to asynchronised nuclear division, as indicated by an increased number of odd nuclei per PV. Asterisks indicate significant differences in asynchronized replication (P<0.05, two tailed Student's t-test). (C) Quantification of the observed defect in IMC formation of the same parasites (−/− parasites not treated with Shld-1; −/+ parasites treated with Shld1 after invasion and +/+, parasites treated with Shld1 before and after invasion). Total number of parasitophorous vacuoles was counted showing a deformed IMC. Parasites treated with 1 µM Shld-1 showed a defect in IMC formation (100%). (4.02 MB TIF) [file ppat.1001029.s003.tif]
