# Supplementary material for: Biogenesis of the Inner Membrane Complex Is Dependent on Vesicular Transport by the Alveolate Specific GTPase Rab11B
Source: PLoS Pathog. 2010 Jul 29;6(7):e1001029. doi: 10.1371/journal.ppat.1001029 (PMC2912401; doi:10.1371/journal.ppat.1001029)
Supplement: Table S1 — List of protein sequences within orthology group OG4_21991 (0.04 MB DOC) [file ppat.1001029.s004.doc]

Table S1. List of Protein Sequences within orthology group OG4_21991

| **Number** | **Accession** | **Taxon** | **Description** |
| --- | --- | --- | --- |
| **1** | [ncan|NCLIV_009790](http://orthomcl.org/cgi-bin/OrthoMclWeb.cgi?rm=sequence&accession=NCLIV_009790&taxon=ncan) | *Neospora caninum* |  |
| **2** | [pber|PB001336.02.0](http://orthomcl.org/cgi-bin/OrthoMclWeb.cgi?rm=sequence&accession=PB001336.02.0&taxon=pber) | *Plasmodium berghei* | GTP-binding protein, putative |
| **3** | [pcha|PCAS_135870](http://orthomcl.org/cgi-bin/OrthoMclWeb.cgi?rm=sequence&accession=PCAS_135870&taxon=pcha) | *Plasmodium chabaudi* | conserved Plasmodium protein, unknwon function |
| **4** | [pfal|MAL13P1.205](http://orthomcl.org/cgi-bin/OrthoMclWeb.cgi?rm=sequence&accession=MAL13P1.205&taxon=pfal) | *Plasmodium falciparum 3D7* | Rab11b, GTPase |
| **5** | [pkno|PKH_121350](http://orthomcl.org/cgi-bin/OrthoMclWeb.cgi?rm=sequence&accession=PKH_121350&taxon=pkno) | *Plasmodium knowlesi strain H* | GTP-binding protein, putative |
| **6** | [pviv|PVX_082950](http://orthomcl.org/cgi-bin/OrthoMclWeb.cgi?rm=sequence&accession=PVX_082950&taxon=pviv) | *Plasmodium vivax SaI-1* | small GTPase rab11b, putative |
| **7** | [pyoe|PY02876](http://orthomcl.org/cgi-bin/OrthoMclWeb.cgi?rm=sequence&accession=PY02876&taxon=pyoe) | *Plasmodium yoelii yoelii str. 17XNL* | small GTPase rab11-related |
| **8** | [tann|TA13860](http://orthomcl.org/cgi-bin/OrthoMclWeb.cgi?rm=sequence&accession=TA13860&taxon=tann) | *Theileria annulata strain Ankara* | GTP-binding protein, Rab-family, putative |
| **9** | [tgon|TGME49_120480](http://orthomcl.org/cgi-bin/OrthoMclWeb.cgi?rm=sequence&accession=TGME49_120480&taxon=tgon) | *Toxoplasma gondii* | Rab 11b, putative |
| **10** | [tpar|XP_765125](http://orthomcl.org/cgi-bin/OrthoMclWeb.cgi?rm=sequence&accession=XP_765125&taxon=tpar) | *Theileria parva strain Muguga* | hypothetical protein TP02_0559 [Theileria parva strain Muguga] |
| **11** | [tthe|41.m00299](http://orthomcl.org/cgi-bin/OrthoMclWeb.cgi?rm=sequence&accession=41.m00299&taxon=tthe) | *Tetrahymena thermophila SB210* | Ras family protein |
| **12** | [bbov|XP_001610116.1](http://orthomcl.org/cgi-bin/OrthoMclWeb.cgi?rm=sequence&accession=XP_001610116.1&taxon=bbov) | *Babesia bovis T2Bo* | Rab11b protein [Babesia bovis T2Bo] |
| **13** | [chom|Chro.70485](http://orthomcl.org/cgi-bin/OrthoMclWeb.cgi?rm=sequence&accession=Chro.70485&taxon=chom) | *Cryptosporidium hominis TU502* | Rab11b |
| **14** | [cmur|CMU_014530](http://orthomcl.org/cgi-bin/OrthoMclWeb.cgi?rm=sequence&accession=CMU_014530&taxon=cmur) | *Cryptosporidium muris RN66* | Rab11b protein, putative |
| **15** | [cpar|cgd7_4380](http://orthomcl.org/cgi-bin/OrthoMclWeb.cgi?rm=sequence&accession=cgd7_4380&taxon=cpar) | *Cryptosporidium parvum Iowa II* | Rablib |
